# Advanced glycation end‐products suppress autophagic flux in podocytes by activating mammalian target of rapamycin and inhibiting nuclear translocation of transcription factor EB

**DOI:** 10.1002/path.5077

**Published:** 2018-04-30

**Authors:** Xingchen Zhao, Yuanhan Chen, Xiaofan Tan, Li Zhang, Hong Zhang, Zhilian Li, Shuangxin Liu, Ruizhao Li, Ting Lin, Ruyi Liao, Qianmei Zhang, Wei Dong, Wei Shi, Xinling Liang

**Affiliations:** ^1^ The Second School of Clinical Medicine Southern Medical University Guangzhou Guangdong PR China; ^2^ Division of Nephrology, Guangdong General Hospital Guangdong Academy of Medical Sciences, Guangdong Provincial Institute of Geriatrics Guangzhou Guangdong PR China; ^3^ Division of Nephrology Zhongshan City People's Hospital, Zhongshan Hospital of Sun Yat‐sen University Zhongshan Guangdong PR China

**Keywords:** diabetic nephropathy, advanced glycation end‐products, podocytes, autophagy, mTOR, transcription factor EB

## Abstract

Insufficient autophagy in podocytes is related to podocyte injury in diabetic nephropathy (DN). Advanced glycation end‐products (AGEs) are major factors of podocyte injury in DN. However, the role and mechanism of AGEs in autophagic dysfunction remain unknown. We investigated autophagic flux in AGE‐stimulated cultured podocytes using multiple assays: western blotting, reverse transcription–quantitative PCR, immunofluorescence staining, and electron microscopy. We also utilized chloroquine and a fluorescent probe to monitor the formation and turnover of autophagosomes. Mice of the db/db strain were used to model diabetes mellitus (DM) with high levels of AGEs. To mimic DM with normal levels of AGEs as a control, we treated db/db mice with pyridoxamine to block AGE formation. AGEs impaired autophagic flux in the cultured podocytes. Compared with db/db mice with normal AGEs but high glucose levels, db/db mice with high AGEs and high glucose levels exhibited lower autophagic activity. Aberrant autophagic flux was related to hyperactive mammalian target of rapamycin (mTOR), a major suppressor of autophagy. Pharmacologic inhibition of mTOR activity restored impaired autophagy. AGEs inhibited the nuclear translocation and activity of the pro‐autophagic transcription factor EB (TFEB) and thus suppressed transcription of its several autophagic target genes. Conversely, TFEB overexpression prevented AGE‐induced autophagy insufficiency. Attenuating mTOR activity recovered TFEB nuclear translocation under AGE stimulation. Co‐immunoprecipitation assays further demonstrated the interaction between mTOR and TFEB in AGE‐stimulated podocytes and in glomeruli from db/db mice. In conclusion, AGEs play a crucial part in suppressing podocyte autophagy under DM conditions. AGEs inhibited the formation and turnover of autophagosomes in podocytes by activating mTOR and inhibiting the nuclear translocation of TFEB. © 2018 The Authors. *The Journal of Pathology* published by John Wiley & Sons Ltd on behalf of Pathological Society of Great Britain and Ireland.

## Introduction

Diabetic nephropathy (DN) is the leading cause of chronic and end‐stage renal disease worldwide 1. It is well known that podocyte injury plays a central part in the proteinuria associated with DN and progression of DN 2. Podocytes are highly differentiated cells with limited capacity to proliferate and regenerate after injury. Thus, self‐repair mechanisms are vital to maintain homeostasis.

Autophagy is an intracellular process that involves the degradation and recycling of defective cytosolic proteins and organelles via lysosomes 3, 4. The autophagic process is executed mainly by a series of proteins that orchestrate the dynamic process of autophagosome formation, fusion of the autophagosomes and lysosomes to form autolysosomes, and the degradation of substrates in autolysosomes. This dynamic flow is called ‘autophagic flux’.

Mammalian target of rapamycin (mTOR) is a serine/threonine kinase that regulates the growth and proliferation of cells. It is the most studied regulator of autophagy; it inhibits autophagy initiation by phosphorylation of autophagy‐related proteins 5. mTOR is hyperactivated in patients with DN 6, and reduction of mTOR activity in animals suffering from diabetes mellitus (DM) suppresses DN development 7, 8.

Transcriptional regulation also plays a vital part in coordinating autophagy 9. Transcription factor EB (TFEB) drives the transcription of various genes involved in the autophagy–lysosomal pathway, including the formation and degradation of autophagosomes as well as lysosomal biogenesis 10. It has been reported that mTOR phosphorylates TFEB and inhibits its activity directly 11, 12, indicating crosstalk between mTOR and TFEB pathways.

Podocytes exhibit a high level of constitutive autophagy, which is crucial for stress adaptation 13. Aberrant accumulation of the autophagy substrate p62/sequestosome 1 in the glomeruli of DM patients with massive proteinuria has been reported 14. In addition, Tagawa *et al* reported that the sera of DM patients and rodents with massive proteinuria caused autophagy insufficiency in cultured podocytes 14, indicating impaired autophagy in the podocytes involved in DN. In streptozotocin (STZ)‐induced DM mice, podocyte autophagy was found to be induced at an early stage following STZ injection but repressed at a late stage 15. The mechanism of action underlying this DM duration‐related reversal of autophagy is not known. After chronic accumulation of advanced glycation end‐products (AGEs), hyperglycemic stress is amplified and a vicious cycle of metabolic disturbances is ignited 16. We hypothesized that AGEs contribute to impairing podocyte autophagy; so we investigated the role and underlying mechanism of AGEs in the autophagic flux of podocytes *in vivo* and *in vitro*.

## Materials and methods

Further details can be found in the supplementary material, Supplementary materials and methods.

### Ethics statement and patient studies

The protocol of this study was approved by the Ethics Committee of Guangdong General Hospital (GDREC2012110; Guangdong, PR China). The study of patients was conducted according to the Helsinki Declaration. Renal tissues from biopsies were obtained with the informed written consent of patients. Normal kidney tissues distal from the tumor margin were obtained from patients with renal cell carcinoma after unilateral nephrectomy. These tissues were confirmed to be normal renal tissue by further pathological examination. Clinical characteristics of the patients are presented in the supplementary material, Table S1.

### Cell treatments

To investigate the role of AGEs, cultured podocytes were exposed to 100 μg/ml AGE–bovine serum albumin (BSA; BioVision, Milpitas, CA, USA) or BSA alone for 72 h unless otherwise noted (supplementary material, Figure S1A). To study autophagosome formation, podocytes were treated with chloroquine (CQ; 10 μm; Sigma‐Aldrich, St Louis, MO, USA) or an equal volume of dimethyl sulfoxide (Sigma‐Aldrich) as vehicle according to our method 17. CQ is a well‐known autophagy inhibitor that blocks the fusion of autophagosomes and lysosomes and interrupts the substrate degradation of autolysosomes 15. Under CQ treatment, the activity of autophagic flux was dependent only on autophagosome formation. To inhibit mTOR activity, cultured podocytes were treated with 50 nm Torin1 or an equal volume of dimethyl sulfoxide as vehicle for 3 h. The treatment time and concentration of CQ or Torin1 were based on the results of our preliminary experiment (supplementary material, Figures S2A and S3A) 17.

### Transfection of adenovirus and small interfering RNAs (siRNAs)

Podocytes were transfected with vector control or flag‐TFEB adenovirus (Hanbio Technology, Shanghai, PR China) for 12 h. Three siRNAs targeting TFEB and control were used (RiboBio, Guangzhou, PR China). The most effective one was selected for final experimentation (supplementary material, Figure S4A–C and Table S2). Podocytes were transfected with the most effective TFEB siRNA or negative control siRNA (scrambled siRNA), using Lipofectamine 2000 (Invitrogen, Carlsbad, CA, USA) according to the manufacturer's protocol. To investigate the autophagic flux, podocytes were transfected with GFP‐mRFP‐LC3 adenovirus as we described previously 17. The GFP‐mRFP‐LC3 protein initially labeled the membranes of autophagosomes and was then delivered to autolysosomes. The GFP fluorescent signal is sensitive to and quenched by the acidic conditions within the lysosomes, but the mRFP fluorescent signal is stable in the lysosome. Co‐localization of GFP and mRFP signals (yellow fluorescence) indicates autophagosomes that have not yet fused with lysosomes. The mRFP signal alone (red fluorescence) without GFP signal indicates autolysosomes 18 (supplementary material, Figure S5).

### ChIP assays

Podocytes treated with BSA or AGE–BSA were used for ChIP assays. The Simple ChIP Enzymatic Chromatin IP Kit (Millipore, Billerica, MA, USA) was used following the manufacturer's protocol. Anti‐TFEB antibody (supplementary material, Table S3) was used to pull down DNA–protein complexes, and goat IgG was used as a control. Purified DNA was quantified using quantitative PCR (qPCR); primer sequences (TaKaRa Biotechnology, Dalian, PR China) are listed in the supplementary material, Table S4.

### Ultrastructural analyses

Transmission electron microscopy (TEM) was used to measure the width of podocyte foot processes and autophagic vacuoles. Kidney tissue specimens from humans or mice or cultured podocytes were fixed using 2.5% glutaraldehyde. Gradient dehydration using alcohol and post‐fixation using 1% osmium tetroxide were completed prior to embedding in resin (Epon 812). Ultrathin sectioning and double staining using uranyl acetate and lead citrate were employed to prepare TEM samples, which were examined using a transmission electron microscope (Tecnai 12; FEI, Lausanne, Switzerland). The width of podocyte foot processes was calculated as described previously 19. The number of autophagosomes in podocytes were counted 20, 21. To examine the ultrastructural features of podocytes, pieces of cortex were fixed in glutaraldehyde and then fixed in osmium tetroxide and sodium cacodylate buffer. After being dehydrated and dried, the material was examined using a scanning electron microscope (Tecnai G^2^ Spirit Twin; FEI).

### Animal studies

Animal experimental procedures were undertaken according to the protocols of the Guangdong Academy of Medical Sciences. Eighteen male C57BL/KsJ db/db mice and six age‐matched non‐diabetic (db/m) mice were purchased from the Model Animal Research Center of Nanjing University (Nanjing, PR China).

To investigate the role of AGEs in the autophagy in podocytes of db/db mice, we generated a DM model with normal levels of AGEs using pyridoxamine (PYR; Sigma‐Aldrich). This agent is a derivative of vitamin B6 and can reduce serum AGE levels by blocking AGE formation with no effect on the blood levels of glucose or lipids 22.

At 8 weeks of age, db/db mice were allocated randomly into db/db (*n* = 6), db/db + PYR (*n* = 6) or db/db + Torin1 (*n* = 6) groups by body weight. PYR was given by gavage (400 mg/kg daily) for 12 weeks. Torin1 (Selleck, Houston, TX, USA), an inhibitor of mTOR, was diluted to 2 mg/ml in 0.15 m NaCl, 5% polyethylene glycol 400, and 5% Tween 20 immediately before injection, as reported previously 23. db/db mice were injected with Torin1 (20 mg/kg, i.p.) daily for the last 10 days before killing.

Fasting blood glucose levels were monitored weekly using a One Touch™ Ultra Glucometer and Test Strips (Lifescan, Milpitas, CA, USA) after 6 h of fasting. AGE levels in the sera of experimental mice at 20 weeks were assessed using an OxiSelect™ Advanced Glycation End Product ELISA kit (Cell Biolabs, San Diego, CA, USA). Body weight was measured every week. For urine collection, once every 2 weeks individual mice were held in a metabolic cage for 24 h. Urinary levels of albumin and creatinine were measured using albumin ELISA (Bethyl Laboratories, Montgomery, TX, USA) and creatinine kits (Cayman Chemical, Ann Arbor, MI, USA) for mice, respectively, according to the manufacturers' instructions. Albuminuria was expressed as the ratio of albumin to creatinine. Mice were anesthetized (pentobarbital, 50 mg/kg, i.p.) before killing, and kidney tissue was collected.

### Isolation of glomeruli

db/db mice (n = 8) and db/db mice treated with PYR (n = 8) were anesthetized and then perfused with 8 × 10^7^ Dynabeads M‐450 (Thermo Scientific, Rockford, IL, USA) through the heart. The kidneys were removed and minced on ice into 1 mm^3^ pieces before digestion by collagenase A (1 mg/ml; Roche Diagnostics, Indianapolis, IN, USA) and deoxyribonuclease I (100 U/ml; Roche Diagnostics) at 37°C for 15 min with gentle agitation. The digested tissue was gently pressed through a 100 μm cell strainer (BD Falcon, Bedford, MA, USA) and then passed through a new strainer without pressing. The filtered tissue was transferred to a cooled tube and centrifuged at 200 × *g* for 5 min. The supernatant was discarded and the pellet was resuspended and transferred to a magnetic particle concentrator, which gathered the glomeruli that contained Dynabeads. The isolated glomeruli were washed at least three times with cold HBSS and then resuspended in lysis buffer (50 mm Tris–HCl, 1% NP‐40, 150 mm NaCl, 15% glycerol, 1 mm EDTA, 1 mm NaF, 1 mm Na_3_VO_4_, pH 7.5, supplemented with protease inhibitors) and homogenized in a Dounce homogenizer at 4°C. After protein extraction, the suspension was centrifuged at 14 000 × *g* for 10 min at 4°C; the pellet containing Dynabeads and insoluble material was discarded; and the supernatant was used for immunoprecipitation assays.

### Statistical analyses

Results are reported as mean ± standard error of the mean (SEM). Statistical analysis was performed with SPSS v21.0 (IBM Corp, Armonk, NY, USA). All experimental procedures were repeated at least three times. Between‐group comparisons were made using Student's *t*‐test. Multiple comparisons were carried out using one‐way analysis of variance (ANOVA) for the cell culture experiments or two‐way ANOVA for the animal experiments. The Student–Newman–Keuls test was used for *post hoc* multiple comparisons. A two‐tailed *p* < 0.05 was considered significant.

## Results

### Impaired podocyte autophagy in patients with DN and the role of AGEs in impaired autophagy in db/db mice

To explore podocyte autophagy in patients with DN, we utilized TEM to examine the number of autophagic vacuoles and immunofluorescent staining to observe microtubule‐associated protein 1 light chain 3 (LC3, a marker for autophagosomes) dots in podocytes.

**Figure 1:**
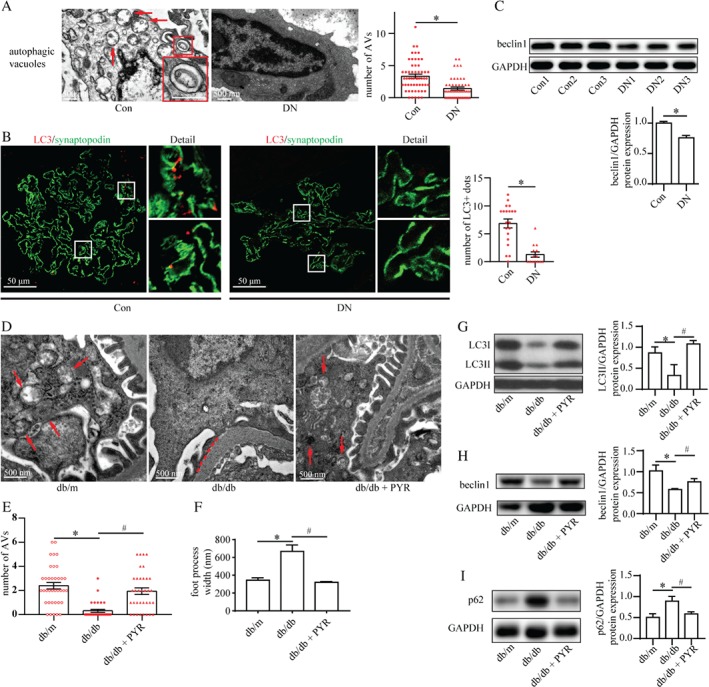
Autophagic insufficiency in podocytes under high AGE conditions. (A) TEM of kidney specimens from control subjects (normal renal tissues from patients with renal cell carcinoma) and patients with diabetic nephropathy (DN). The number of autophagic vacuoles (AVs; arrows) was reduced in podocytes from patients with DN (means ± SEM, n = 4 for control subjects, n = 5 for patients with DN; 51–57 images were selected from each group). Student's t‐test; *p < 0.05 versus control group. Scale bar = 500 nm. (B) Immunofluorescence staining for LC3 (red) and synaptopodin (green) in the renal cortex from control subjects and patients with DN. Quantification of the number of LC3‐positive dots (yellow) indicated a reduction in the number of autophagosomes in podocytes from patients with DN (means ± SEM, n = 3; 14–20 images from each group). Student's t‐test; *p < 0.05 versus control. Scale bar = 50 μm. (C) Western blot assay of beclin1 expression in the renal cortex from control subjects and patients with DN. Beclin1 expression normalized against GAPDH was reduced in the renal cortex from patients with DN (means ± SEM, n = 3). Student's t‐test; *p < 0.05 versus control. (D–F) TEM of the renal cortex from db/m mice treated with vehicle (db/m, representing non‐diabetic control), db/db mice treated with vehicle (db/db, representing diabetic mice with a high AGE level), and db/db mice treated with PYR (db/db + PYR, representing diabetic mice with a normal AGE level). PYR recovered the reduced number of AVs in podocytes from db/db mice (E) (means ± SEM, n = 4; 32–36 images from each group). An increased width of podocyte foot processes (dashed line) in db/db mice was prevented by PYR treatment (F) (means ± SEM, n = 4; 32–36 images from each group). Two‐way ANOVA, followed by post hoc Student–Newman–Keuls test; *p < 0.05 versus db/m mice; ^#^ p < 0.05 versus db/db mice. Scale bar = 500 nm. (G–I) Western blots for expression of LC3II (G), beclin1 (H), and p62 (I) from the renal cortex in db/m, db/db, and db/db + PYR mice. Downregulation of the expression of LC3II and beclin1 accompanied by upregulation of p62 expression indicated the inhibition of autophagic flux in db/db mice, which was restored by PYR treatment (means ± SEM, n = 4). Two‐way ANOVA, followed by post hoc Student–Newman–Keuls test; *p < 0.05 versus db/m mice; ^#^ p < 0.05 versus db/db mice.

**Figure 2:**
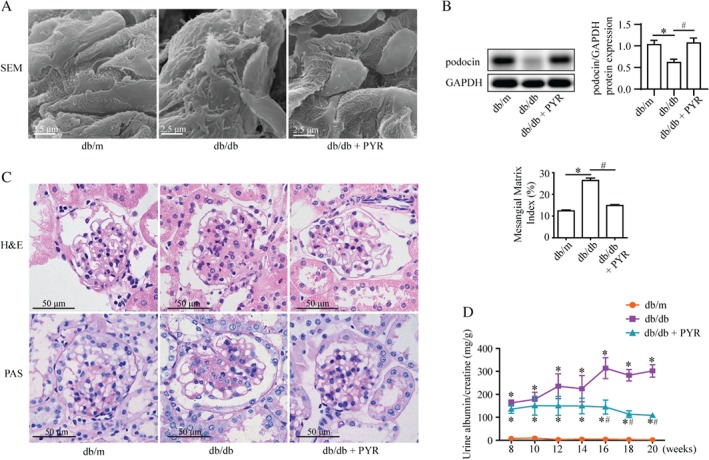
AGEs induce podocyte injury and renal tissue damage in db/db mice. (A) Representative scanning electron micrographs of the renal cortex from the indicated mice. PYR protected against abnormal podocytes in db/db mice. Scale bar = 2.5 μm. (B) Western blotting of podocin expression from the renal cortex suggesting that PYR recovered the decreased podocin level in db/db mice (mean ± SEM, n = 3). Two‐way ANOVA, followed by post hoc Student–Newman–Keuls test; *p < 0.05 versus db/m mice; ^#^ p < 0.05 versus db/db mice. (C) H & E and periodic acid–Schiff (PAS) staining of renal tissues. The mesangial matrix index indicated greater mesangial expansion in db/db mice than in non‐diabetic controls, whereas PYR reduced this mesangial expansion. Scale bar = 50 μm (means ± SEM, n = 5; 30 images from each group). Two‐way ANOVA, followed by post hoc Student–Newman–Keuls test; *p < 0.05 versus db/m mice; ^#^ p < 0.05 versus db/db mice. (D) Urinary albumin excretion (mg/g creatinine) of db/m, db/db, and db/db + PYR mice. Urine samples were collected in a metabolic cage at 8, 10, 12, 14, 16, 18, and 20 weeks of age. PYR attenuated the increased albuminuria in db/db mice at 16–20 weeks (n = 3). Repeated measures ANOVA followed by post hoc Student–Newman–Keuls test; *p < 0.05 versus db/m mice; ^#^ p < 0.05 versus db/db mice.

We found fewer autophagic vacuoles and LC3 dots in podocytes from patients with DN compared with the control (Figure 1A, B). Western blotting also showed a reduction of beclin1 (a marker for autophagy initiation) levels in the renal cortex from patients with DN (Figure 1C). One study observed abnormal accumulation of p62 (indicative of insufficient autophagy) in the glomeruli of patients with DN 24. Consistent with that finding, our findings further clarified insufficient autophagy in podocytes in DN. Moreover, db/db mice treated with or without PYR mimicked a hyperglycemic and hyperlipidemic DM condition with normal or high levels of AGEs, respectively (supplementary material, Figure S6A–C). The db/db mice exhibited a significant reduction in the number of autophagic vacuoles in podocytes by TEM, compared with db/db mice treated with PYR and db/m control mice (Figure 1D, E). This impaired autophagy in podocytes was further supported by the results from western blotting of decreased levels of LC3II and beclin1 and increased levels of p62 (Figure 1G–I). Also, db/db mice exhibited significant effacement of podocyte foot processes and increased slit width compared with db/db mice treated with PYR and control mice (Figures 1D, F and 2A). AGE‐related podocyte injury was revealed by downregulation of the expression of podocin, an essential cytoskeletal protein of podocytes (Figure 2B). In addition, db/db mice exhibited glomerular hypertrophy, glomerular basement membrane thickening, and mesangial expansion relative to db/db mice treated with PYR and db/m control mice (Figure 2C). Also, the urinary albumin/creatinine ratio was significantly higher in the db/db group than in db/db mice treated with PYR and db/m control mice (Figure 2D). Taken together, these results indicate that the high AGE levels under DM conditions inhibited autophagy and led to podocyte damage and renal pathologic injury *in vivo*.

### AGEs interrupted autophagosome formation and turnover

**Figure 3:**
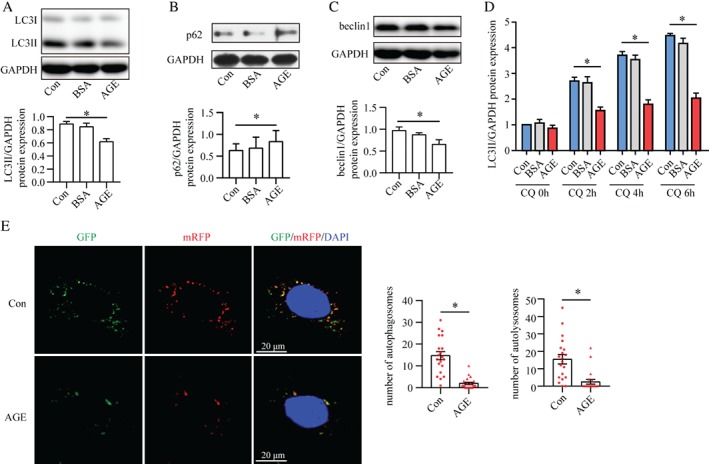
AGEs impair the formation and turnover of autophagosomes. (A–C) Western blots of the expression of LC3II (A), p62 (B), and beclin1 (C) in cultured podocytes treated with vehicle (Con), BSA or AGE–BSA (AGE). Autophagic flux was inhibited in AGE‐stimulated podocytes compared with Con and BSA, as demonstrated by the reduction in expression of LC3II and belin1 as well as the accumulation of p62 (means ± SEM, n = 3–4). One‐way ANOVA, followed by post hoc Student–Newman–Keuls test; *p < 0.05 versus control. (D) AGEs reduced chloroquine (CQ)‐induced LC3II accumulation according to western blotting, suggesting blockade of autophagosome formation (mean ± SEM, n = 3). One‐way ANOVA, followed by post hoc Student–Newman–Keuls test; *p < 0.05 versus control. (E) Confocal laser scanning microscopy images of podocytes transfected with the tandem GFP‐mRFP‐LC3 adenovirus. The number of autophagosomes (yellow) and autolysosomes (red) was reduced under AGE stimulation, indicating dual suppression of the formation and turnover of autophagosomes (mean ± SEM, n = 3; 20–21 cells from each group). Student's t‐test; *p < 0.05 versus control. Scale bar = 20 μm.

To understand the role of AGEs in the absence of high glucose levels in autophagic activity, we stimulated cultured podocytes with AGE–BSA. Under this condition, western blotting showed that the biosynthesis of LC3II protein and turnover of p62 were blocked, indicating inhibited autophagy (Figure 3A, B). We then investigated the effect of AGEs on autophagic flux. Levels of the autophagy‐initiation protein beclin1 were decreased in AGE‐stimulated podocytes (Figure 3C). To exclude a potential effect of autophagosome turnover on the decreased numbers of autophagosomes, we blocked autophagosome turnover using the lysosome inhibitor CQ. The accumulation of LC3II induced by CQ was reduced by AGEs plus CQ stimulation (Figure 3D and supplementary material, Figure S2A, B). In addition, in Ad‐GFP‐mRFP‐LC3‐transfected podocytes, the number of autophagosomes (yellow puncta) was decreased under AGE stimulation. These results demonstrate that AGEs blocked the formation of autophagosomes. Moreover, in Ad‐GFP‐mRFP‐LC3‐transfected podocytes, the number of autolysosomes (red puncta) was also decreased under AGE stimulation (Figure 3E), indicating that AGEs also interrupted autophagosome turnover in autophagic flux.

### AGEs reduced podocyte autophagy through mTOR activation

**Figure 4:**
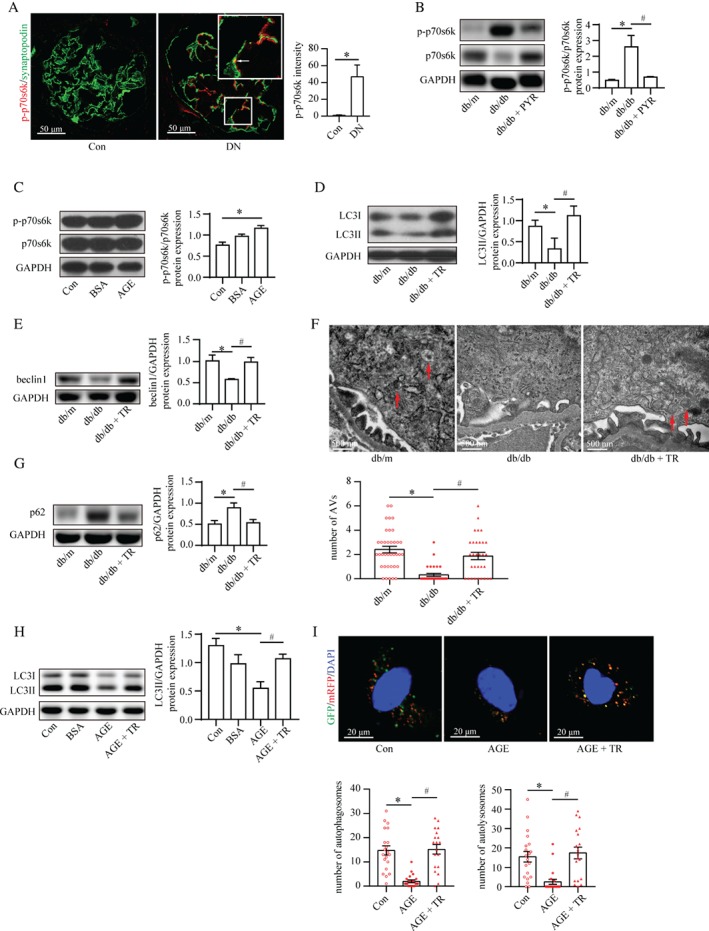
mTOR activation mediates AGE‐stimulated autophagy impairment. (A) Immunofluorescence staining for phospho‐p70s6k (p‐p70s6k, a marker of mTOR activity; red) and synaptopodin (green) from controls and patients with DN. The p‐p70s6k expression in podocytes (arrows) was enhanced in patients with DN (mean ± SEM, n = 3; 12 images from each group). Student's t‐test; *p < 0.05 versus control group. Scale bar = 50 μm. (B) The ratio of p‐p70s6k to p70s6k was increased in db/db mice compared with control, indicating mTOR hyperactivation, by western blotting, which was recovered to normal levels by PYR (mean ± SEM, n = 4). Two‐way ANOVA, followed by post hoc Student–Newman–Keuls test; *p < 0.05 versus db/m mice; ^#^ p < 0.05 versus db/db mice. (C) The same pattern of mTOR activity was observed in AGE‐stimulated podocytes (mean ± SEM, n = 3). One‐way ANOVA, followed by post hoc Student–Newman–Keuls test; *p < 0.05 versus control. (D, E) Torin1 restored the attenuated level of LC3II (D) and beclin1 (E) in db/db mice according to western blotting (mean ± SEM, n = 3–4). Two‐way ANOVA, followed by post hoc Student–Newman–Keuls test; *p < 0.05 versus db/m mice; ^#^ p < 0.05 versus db/db mice. (F) TEM showed that Torin1 recovered the reduced number of autophagic vacuoles (AVs) in podocytes from db/db mice (mean ± SEM, n = 4; 31–37 images from each group). Two‐way ANOVA, followed by post hoc Student–Newman–Keuls test; *p < 0.05 versus db/m mice; ^#^ p < 0.05 versus db/db mice. Scale bar = 500 nm. (G) Torin1 reduced the p62 level in db/db mice by western blotting (mean ± SEM, n = 4). Two‐way ANOVA, followed by post hoc Student–Newman–Keuls test; *p < 0.05 versus db/m mice; ^#^ p < 0.05 versus db/db mice. (H) Torin1 restored the reduction of LC3II expression in AGE‐stimulated podocytes by western blotting (mean ± SEM, n = 3). One‐way ANOVA, followed by post hoc Student–Newman–Keuls test; *p < 0.05 versus control; ^#^ p < 0.05 versus AGE. (I) Confocal laser scanning microscopy images of podocytes transfected with the tandem GFP‐mRFP‐LC3 adenovirus. Torin1 recovered the reduced number of autophagosomes (yellow) and autolysosomes (red) in AGE‐stimulated podocytes (mean ± SEM, n = 3; 19–21 cells from each group). One‐way ANOVA, followed by post hoc Student–Newman–Keuls test; *p < 0.05 versus control; ^#^ p < 0.05 versus AGE. Scale bar = 20 μm.

Consistent with previous studies 6, mTOR was hyperactivated in podocytes from patients with DN compared with the control (Figure 4A). The mTOR activity was also upregulated in the renal cortex from db/db mice and in AGE‐induced cultured podocytes (Figure 4B, C). Next, we utilized Torin1, an mTOR inhibitor (supplementary material, Figure S3A, B), to elucidate whether this mTOR activation mediated AGE‐stimulated autophagy inhibition in db/db mice and in AGE‐stimulated cultured podocytes. The levels of LC3II and beclin1, and the number of autophagic vacuoles were found to have recovered in db/db mice treated with Torin1 compared with those in db/db mice (Figure 4D–F). In parallel, Torin1 markedly reduced p62 levels in db/db mice (Figure 4G). Furthermore, in AGE‐stimulated cultured podocytes, Torin1 restored the reduction of LC3II levels as well as the number of autophagosomes and autolysosomes (Figure 4H, I). These data indicate that AGEs impaired autophagic flux through mTOR activation.

### TFEB regulated podocyte autophagy

The above‐mentioned results indicated that AGEs suppressed the formation and turnover of autophagosomes. Because TFEB, the ‘master regulator’ of autophagy, controls multiple steps of the autophagic pathway 10, we speculated that it might participate in AGE‐regulated autophagy.

**Figure 5:**
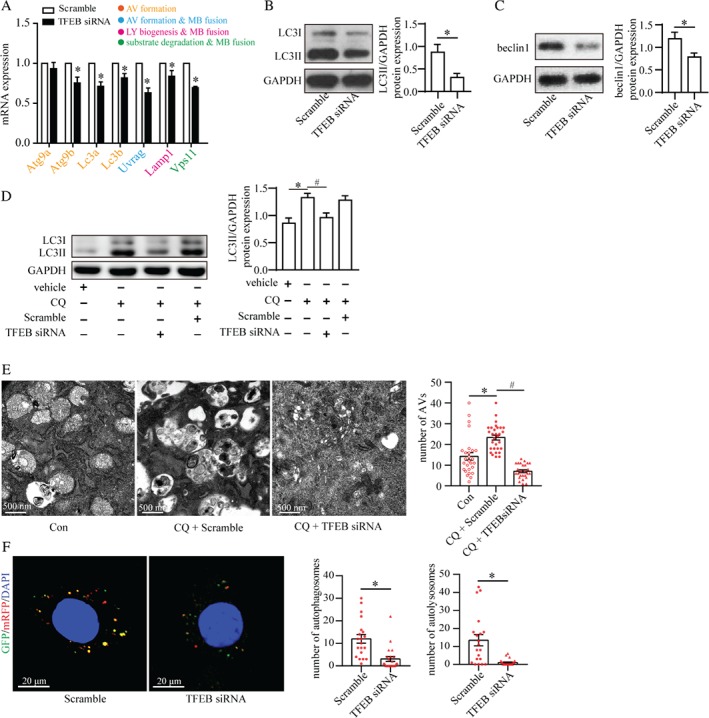
Role of TFEB in autophagic flux in podocytes. (A) Reverse transcription–quantitative PCR (RT‐qPCR) analyses of the expression of TFEB target genes in podocytes treated with scrambled siRNA (Scramble) or TFEB siRNA. The colors represent the functions of genes in regulating autophagy as indicated. TFEB siRNA reduced the mRNA expression of the genes involved in the formation and turnover of autophagosomes (mean ± SEM, n = 3). Student's t‐test; *p < 0.05 versus scrambled siRNA. AV: autophagic vacuoles; MB: membrane; LY: lysosome. (B, C) TFEB siRNA decreased the level of LC3II (B) and beclin1 (C) according to western blotting (mean ± SEM, n = 3). Student's t‐test; *p < 0.05 versus scrambled siRNA. (D) TFEB siRNA reduced chloroquine (CQ)‐induced LC3II accumulation, suggesting the importance of TFEB in autophagosome formation (mean ± SEM, n = 3). One‐way ANOVA, followed by post hoc Student–Newman–Keuls test; *p < 0.05 versus vehicle; ^#^ p < 0.05 versus CQ. (E) TEM revealed that the number of accumulated autophagic vacuoles (AVs) in CQ‐stimulated podocytes was reduced by TFEB siRNA (mean ± SEM, n = 3; 26–30 images from each group). One‐way ANOVA, followed by post hoc Student–Newman–Keuls test; *p < 0.05 versus control; ^#^ p < 0.05 versus CQ + scrambled siRNA. Scale bar = 500 nm. (F) Confocal laser scanning microscopy of podocytes transfected with the tandem GFP‐mRFP‐LC3 adenovirus. TFEB siRNA reduced the number of autophagosomes (yellow) and autolysosomes (red) (mean ± SEM, n = 3; 20–22 cells from each group). Student's t‐test; *p < 0.05 versus scrambled siRNA. Scale bar = 20 μm.

We first explored the effect of TFEB in podocyte autophagy. The mRNA levels of TFEB‐regulated autophagy genes were decreased significantly in the TFEB‐silenced podocytes involved in autophagosome formation (*Atg9b*, *Lc3a*, *Lc3b*, and *Uvrag*), membrane fusion (*Uvrag*, *Lamp1*, and *Vps11*), lysosome biogenesis (*Lamp1*), and substrate degradation (*Vps11*) (Figure 5A). In addition, TFEB knockdown reduced the protein levels of LC3II and beclin1 significantly (Figure 5B, C). Moreover, TFEB siRNA decreased the CQ‐induced autophagosome accumulation in cultured podocytes significantly (Figure 5D, E). TFEB silencing also decreased the number of autophagosomes (yellow puncta) and autolysosomes (red puncta) in Ad‐GFP‐mRFP‐LC3‐transfected podocytes (Figure 5F). This autophagic disturbance by TFEB silencing was in accordance with the reduced protein levels of two markers of intact podocytes: podocin and synaptopodin (supplementary material, Figure S4D, E). TFEB knockdown was also associated with an indicator of increased podocyte injury: urokinase‐type plasminogen activator receptor (uPAR) 25 (supplementary material, Figure S4F). Taken together, the results indicate that TFEB played a crucial part in maintaining autophagy and homeostasis in podocytes.

### AGEs interrupted autophagic flux by inhibiting nuclear translocation of TFEB

**Figure 6:**
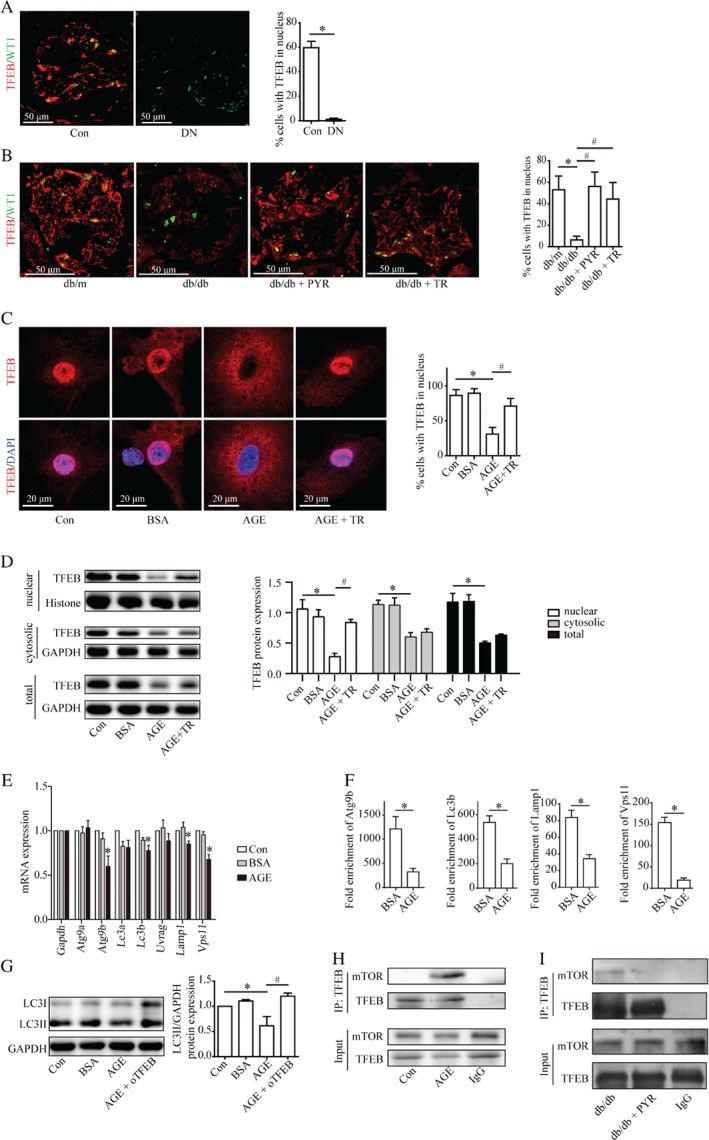
TFEB inhibition mediates the impairment of autophagy in AGE‐stimulated podocytes and crosstalk between mTOR and TFEB. (A) Immunofluorescence staining for TFEB (red) and WT‐1 (green) of kidney specimens from controls and patients with DN. The nuclear localization of TFEB in podocytes was inhibited in patients with DN (mean ± SEM, n = 3; ten images from each group). Student's t‐test; *p <0.05 versus control group. Scale bar = 50 μm. (B) PYR or Torin1 restored the inhibited nuclear localization of TFEB in podocytes in db/db mice according to immunofluorescence analyses (mean ± SEM, n = 3; ten images from each group). Two‐way ANOVA, followed by post hoc Student–Newman–Keuls test; *p < 0.05 versus db/m mice; ^#^ p < 0.05 versus db/db mice. (C) TFEB was overexpressed in podocytes transfected with the recombinant Flag‐TFEB adenovirus. Torin1 promoted the nuclear localization of TFEB which was blocked in AGE‐stimulated podocytes according to immunofluorescence analyses (mean ± SEM, n = 3; 10–16 images from each group). One‐way ANOVA, followed by post hoc Student–Newman–Keuls test; *p < 0.05 versus control; ^#^ p < 0.05 versus AGE. Scale bar = 20 μm. (D) Western blots of TFEB expression in cultured podocytes. The nuclear, cytosolic, and total TFEB expression were decreased under AGE stimulation for 72 h. Torin1 recovered the nuclear TFEB expression which was reduced in AGE‐stimulated podocytes (mean ± SEM, n = 3). One‐way ANOVA, followed by post hoc Student–Newman–Keuls test; *p < 0.05 versus control; ^#^ p < 0.05 versus AGE. (E) Reverse transcription–quantitative PCR (RT‐qPCR) analyses of the expression of TFEB target genes in podocytes: mRNA levels of TFEB‐targeted genes (Atg9b, Lc3b, Lamp1, and Vps11) were decreased in AGE‐stimulated podocytes (mean ± SEM, n = 3). One‐way ANOVA, followed by post hoc Student–Newman–Keuls test; *p < 0.05 versus control. (F) Chromatin immunoprecipitation analyses (ChIP) of the binding of TFEB to the promoter of Atg9b, Lc3b, Lamp1, and Vps11 using an antibody to TFEB. IgG was used as a negative control. Quantitative PCR was conducted to measure the immunoprecipitated DNA using a promoter‐specific primer. Binding of Atg9b, Lc3b, Lamp1, and Vps11 was suppressed under AGE stimulation. Fold enrichment = [% (ChIP/Input)]/[% (Negative control/Input)] (mean ± SEM, n = 3). Student's t‐test; *p < 0.05 versus BSA. (G) TFEB overexpression (oTFEB) restored the decreased LC3II level in AGE‐stimulated podocytes (mean ± SEM, n = 3). One‐way ANOVA, followed by post hoc Student–Newman–Keuls test; *p < 0.05 versus Con; ^#^ p < 0.05 versus AGE. (H) Lysates from cultured podocytes treated with or without AGEs were subjected to immunoprecipitation using an anti‐TFEB antibody or IgG antibody followed by western blotting for mTOR. Input proteins were detected with anti‐TFEB and anti‐mTOR antibodies. AGEs promoted the interaction between TFEB and mTOR. (I) Lysates from glomeruli in db/db mice or db/db + PYR mice were subjected to immunoprecipitation using an anti‐TFEB antibody or IgG antibody followed by western blotting for mTOR. Input proteins were detected with anti‐TFEB and anti‐mTOR antibodies. TFEB interacted with mTOR in db/db mice, which was blocked by PYR.

Nuclear TFEB localization was decreased in podocytes from patients with DN (Figure 6A) and db/db mice (Figure 6B). Considering the discrepancy of TFEB expression between glomeruli (Figure 6B) and renal cortex (supplementary material, Figure S7A) in db/db mice, which might be attributed to the increased TFEB in the renal tubules (supplementary material, Figure S7B), we then investigated the effect of AGEs on TFEB in cultured podocytes.

After AGE stimulation for 48 h, nuclear TFEB expression was attenuated while cytosolic and total TFEB expression were not (Figure 6C and supplementary material, Figure S7C) until 72 h (Figure 6D). In accord with the decreased nuclear TFEB, AGE stimulation also downregulated the mRNA levels of TFEB target genes, especially Atg9b, Lc3b, Lamp1, and Vps11 (Figure 6E). The results of ChIP assays further demonstrated that AGEs impeded TFEB from binding to the promoters of Atg9b, Lc3b, Lamp1, and Vps11 (Figure 6F and supplementary material, Figure S8A). Furthermore, overexpression of TFEB restored the reduced LC3II level in AGE‐stimulated podocytes in vitro (Figure 6G and supplementary material, Figure S4G, H). These data demonstrate AGEs reduced podocyte autophagy in a transcription‐dependent way, which was mediated by suppressing TFEB.

### Crosstalk between mTOR and TFEB under the stimulation of AGEs

It has been reported that Torin1 (but not rapamycin) can recover TFEB activity through mTOR inhibition 11. The former is a novel agent with stronger capacity to inhibit mTOR activity compared with rapamycin 11, 26.

First, we evaluated these two mTOR inhibitors and further investigated the interplay between mTOR and TFEB. Rapamycin had no effect on TFEB distribution (supplementary material, Figure S9A), whereas Torin1 recovered nuclear TFEB expression in db/db mice (Figure 6B and supplementary material, Figure S7A) and in AGE‐stimulated cultured podocytes (Figure 6C, D and supplementary material, Figure S7C). Co‐immunoprecipitation (Co‐IP) results showed that TFEB could interact with mTOR in AGE‐stimulated podocytes but not in the control (without AGE stimulation), as shown in Figure 6H. mTOR also interacted with TFEB in the glomeruli of db/db mice, but not in those treated with PYR (Figure 6I). These results reveal that AGEs impaired autophagic flux in podocytes (at least in part) through the interaction between hyperactive mTOR and TFEB, followed by inhibition of TFEB nuclear translocation.

## Discussion

Insufficient autophagy in podocytes is a characteristic of DM patients and animals. Podocyte‐specific autophagy deficiency aggravates podocyte injury in DM mice 15, 24. Thus, improving autophagy in podocytes is a potential treatment strategy for DN.

Tagawa *et al* found that sera from DM animals impaired autophagy in podocytes *in vitro* 24. However, the key pathogenic factors in sera remain unclear. It is intuitive to use a high glucose‐induced model to mimic the DM condition to study autophagy because high glucose levels are key characteristics of DM. However, negative 24, suppressive 27, 28, 29, and stimulatory 30, 31, 32 effects of high glucose on podocyte autophagy have been reported. These contrasting results indicate that other interfering factors may be involved in regulating autophagy.

In STZ‐induced DM mice, staining for the autophagy marker LC3 in podocytes was found to be increased 4 weeks post‐STZ injection but decreased to below normal conditions 8 weeks post‐STZ injection 15. This DM duration‐related reversal of autophagy prompted us to speculate the potential role of AGEs (products of long‐term glycation stress) in podocyte autophagy.

We compared db/db mice with high or relatively normal AGE levels and also stimulated cultured podocytes with AGE–BSA or BSA. We monitored autophagic flux by multiple assays: western blotting, TEM, tandem probe GFP‐mRFP‐LC3, and RT‐qPCR. Our results demonstrate, for the first time, that AGEs blocked podocyte autophagic flux. Importantly, blockade of AGE accumulation in PRY‐treated db/db mice could recover podocyte autophagy. Because the glucose level in these PRY‐treated db/db mice remained high, this result implies the importance of AGEs in autophagy.

We studied further the role of AGEs in the formation and turnover of autophagosomes. AGEs decreased expression of the markers of autophagosome formation (LC3II, beclin1) *in vitro*. This suppression was confirmed further using the lysosomal inhibitor CQ and tandem probe GFP‐mRFP‐LC3. In db/db mice, expression of LC3II and beclin1 in kidney cortices and autophagosome number in podocytes were decreased. This downregulation of LC3II expression in db/db mice is not in accordance with a previous study suggesting upregulation of LC3 expression in STZ‐induced DM mice 4 weeks after STZ injection 15. Considering that AGEs are formed by long‐term hyperglycemia *in vivo*, 4 weeks of STZ injection may be too short for AGE accumulation. Conversely, db/db mice were under chronic hyperglycemia and had 1.7‐fold more AGEs than control mice (supplementary material, Figure S6A). Our results are in accordance with data showing decreased LC3 expression in STZ‐induced DM mice 8 weeks after STZ injection 15 when the serum concentration of AGEs increased 33. We also found a decreased number of autolysosomes under AGE stimulation using GFP‐mRFP‐LC3 probes. This impaired autophagosome turnover was supported by the decreased transcript levels for the lysosomal gene *Lamp1* and is consistent with Tagawa *et al*'s report 24. Therefore, AGEs disrupted multiple steps of autophagic flux in podocytes.

The hyperactivation of mTOR in podocytes is a crucial step in the development of DN 7, but the role of hyperactive mTOR in podocyte autophagy under DM conditions had not been explored. We identified that mTOR activation was accompanied by autophagy dysfunction under a high‐AGE condition. In addition, pharmacologic repression of mTOR restored the impaired autophagy. These results indicate that activated mTOR‐induced autophagic dysfunction might be responsible for the podocyte injury observed in the high‐AGE condition.

Besides post‐translational modulation by mTOR, autophagy can also be regulated by transcription factors such as TFEB 10. TFEB knockdown led to a deficiency in podocyte autophagy in the present study. Using a CQ‐induced model and tandem probe GFP‐mRFP‐LC3, we observed that TFEB knockdown inhibited the formation and turnover of autophagosomes, as well as reducing the mRNA levels of target genes involved in the various steps of autophagic flux. These findings indicate the importance of TFEB in maintaining podocyte autophagy. In addition, TFEB knockdown led to decreases in the levels of podocin and synaptopodin, and an increase in uPAR expression, indicating the importance of TFEB for the skeleton and stability of podocytes. Previous studies do not support the notion that insufficient autophagy has a role in skeletal disturbance in podocytes 13. Also, TFEB is a multifunctional transcription factor 34. Therefore, whether TFEB maintains podocyte homeostasis through an autophagic pathway requires further investigation.

TFEB is a therapeutic target for lysosomal‐storage diseases 35, 36. In the present study, AGEs prevented TFEB nuclear translocation, resulting in halting the binding of TFEB to the promoters of autophagic target genes and decreasing their transcription. Thus, DN is also associated with TFEB dysfunction. Conversely, TFEB overexpression recovered autophagy, confirming that TFEB inhibition mediated the AGE‐stimulated autophagy impairment in podocytes. These findings indicate the potential value of TFEB as a therapeutic target in autophagy‐dysfunction diseases (including DN).

Recently, studies have unveiled the crucial role of mTOR as a TFEB regulator. However, the effect of mTOR on TFEB is significantly different. Brugarolas and co‐workers proposed that in Tsc2 knockout (mTOR constitutive activation) cells, TFEB is transported into the nucleus 37. Conversely, Puertollano and co‐workers and Ballabio and co‐workers observed that under nutrient‐rich conditions, active mTOR induced sequestration of TFEB in the cytosol, whereas under starvation or lysosome disruption, mTOR inactivation triggered TFEB transport into the nucleus and transcription of autophagy‐associated genes 11, 12. These converse results suggested that the different effects of mTOR on TFEB may depend on different types of stimulation signals. In agreement with the work of Puertollano and co‐workers and Ballabio and co‐workers, we found that under AGE stimulation, activated mTOR induced TFEB accumulation in the cytosol. We further found (by the ChIP assay) that this AGE‐mediated TFEB sequestration in the cytosol contributed to TFEB inactivation to decrease the transcription of autophagic target genes. Thus, our results confirm some of the results from previous studies and extend our understanding of the mTOR–TFEB pathway in response to glycation stress.

Nuclear localization of TFEB can be regulated by multiple serine/threonine kinases under different conditions, including mTOR, extracellular regulated kinase 10, glycogen synthase kinase‐3 38, and Akt (protein kinase B) 39. Considering that mTOR is a key autophagy regulator and is implicated in DN pathogenesis, we focused only on the interplay between mTOR and TFEB under AGE stimulation, which was confirmed by Co‐IP assays. Whether the other serine/threonine kinases participated in TFEB modulation in DN needs to be studied. In addition, the potential mechanism of the decrease of TFEB expression under 72 h AGE stimulation also merits further investigation.

In conclusion, we unveiled a crucial role of AGEs in suppressing podocyte autophagy under DM conditions. We also found that TFEB is essential for autophagy and homeostasis in podocytes. AGEs inhibited the formation and turnover of autophagosomes in podocytes by activating mTOR and inhibition of the nuclear translocation of TFEB (supplementary material, Figure S10).

## Author contributions statement

XL and WS conceived the experiments. XZ, YC, and WS analyzed the data. XZ, YC, and XL wrote the manuscript. All the authors were involved in carrying out the experiments and had final approval of the submitted and published versions.

**SUPPLEMENTARY MATERIAL ONLINE**

**Supplementary materials and methods**

**Figure S1.** Role of AGEs in LC3II expression

**Figure S2.** AGEs block autophagosome formation

**Figure S3.** Torin1 reduces mTOR activity

**Figure S4.** Efficiency of TFEB interference and podocyte injury by TFEB knockdown

**Figure S5.** The principle of the GFP‐mRFP‐LC3 fluorescent probe

**Figure S6.** Effect of pyridoxamine (PYR) on blood levels of glucose, AGEs, and lipid parameters in experimental mice

**Figure S7.** Change in TFEB expression in db/db mice and AGE‐induced podocytes

**Figure S8.** DNA electrophoretogram

**Figure S9.** Role of rapamycin in TFEB nuclear expression

**Figure S10.** The proposed role of AGEs in suppressing podocyte autophagy

**Table S1.** Clinical characteristics in control subjects and patients with diabetic nephropathy

**Table S2.** SiRNA sequences

**Table S3.** Antibody information

**Table S4.** Primers used for ChIP assays

**Table S5.** Primers used for RT‐qPCR

## Supporting information


**Appendix S1.** Supplementary materials and methods

**Figure S1. Role of AGEs on LC3II expression.** (A) Cultured podocytes were treated with BSA or AGE‐BSA (AGE) for the indicated time. Western blotting indicated that AGE inhibited LC3II expression at 72 h (mean ± SEM, n = 3) One way ANOVA, followed by post hoc Student–Newman–Keuls test. *P < 0.05 versus control.
**Figure S2. AGEs block autophagosome formation.** AGEs decreased chloroquine (CQ)‐induced LC3II accumulation as shown by western blotting (A) and reduced CQ‐induced autophagosome accumulation on TEM (B) (means ± SEM, n = 3, and 27–30 images from each group). One‐way ANOVA, followed by post hoc Student–Newman–Keuls test. *P <0.05 versus control, #P <0.05 versus CQ.
**Figure S3. Torin1 reduces mTOR activity.** (A) Western blot assays show reduction of the p‐p70s6k to p70s6k ratio at 3 h in cultured podocytes by various Torin1 (TR) concentrations (mean ± SEM, n = 3). One‐way ANOVA, followed by post hoc Student–Newman–Keuls test. *P <0.05 versus control. (B) Western blot assays of p‐p70s6k and p70s6k expression from the renal cortex showed that the p‐p70s6k to p70s6k ratio was increased in db/db mice (mean ± SEM, n = 4). Two‐way ANOVA, followed by post hoc Student–Newman–Keuls test. *P <0.05 versus control, #P <0.05 versus db/db mice.
**Figure S4. Efficiency of TFEB interference and podocyte injury by TFEB knockdown.** (A) Cultured podocytes were transfected with three siRNAs designed to target TFEB or scrambled siRNA. RT‐qPCR showed that TFEB mRNA expression was significantly downregulated by TFEB siRNA but not TFEB siRNA‐2 or TFEB siRNA‐3 (means ± SEM, n = 3). One‐way ANOVA, followed by post hoc Student–Newman–Keuls test, *P <0.05 versus control. (B) Western blot assay results showed that TFEB siRNA significantly reduced TFEB protein expression (mean ± SEM, n = 3). One‐way ANOVA, followed by post hoc Student–Newman–Keuls test. *P <0.05 versus control. (C) Western blot assays showed that TFEB siRNA significantly reduced nuclear TFEB expression (mean ± SEM, n = 3). One‐way ANOVA, followed by post hoc Student–Newman–Keuls test, *P <0.05 versus control. (D–F) TFEB siRNA significantly downregulated the expression of podocin (D) and synaptopodin (E) proteins, which are markers of intact podocytes, and (F) upregulated the expression of urokinase‐type plasminogen activator receptor (uPAR) a marker of podocyte injury. (mean ± SEM, n = 3) Student's t‐test, *P <0.05 versus scrambled siRNA. (G) Cultured podocytes were transfected with and empty vector control or flag‐TFEB adenovirus (oTFEB). Expression of TFEB mRNA was significantly increased by oTFEB (mean ± SEM, n = 3). One‐way ANOVA, followed by post hoc Student–Newman–Keuls test, *P <0.05 versus control. (H) Western blot assays show that TFEB protein expression was upregulated by oTFEB.
**Figure S5. The principle of the GFP‐mRFP‐LC3 fluorescent probe.**
**Figure S6. Effect of pyridoxamine (PYR) on blood levels of glucose, AGEs, and lipid parameters in experimental mice.** Serum AGEs (A), blood glucose (A), total cholesterol (B) and triglycerides (TRIGs) (C) were increased in db/db mice compared with db/m control mice. PYR reduced AGEs without affecting blood glucose, total cholesterol and TRIGs (mean ± SEM, n = 3). Two‐way ANOVA, followed by post hoc Student–Newman–Keuls test, *P <0.05 versus db/m mice, #P <0.05 versus db/db mice. NS: no significance.
**Figure S7. Change in TFEB expression in db/db mice and AGEs‐induced podocytes. (A)** TFEB expression in the nuclear and cytosolic fractions of mouse renal cortex was assayed by western blotting. Both PYR or torin1 reduced the expression of nuclear TFEB in db/db mice and increased cytosolic expression (mean ± SEM, n = 4). One‐way ANOVA, followed by post hoc Student–Newman–Keuls test, *P < 0.05 versus db/m mice, #P <0.05 versus db/db mice. (B) Immunofluorescence staining of TFEB (red) in the renal cortex of db/db mice and db/m mice showed that TFEB was expressed not only in podocytes but also in other renal resident cells (dashed frame). TFEB expression in the renal tubules tended to increase under diabetic conditions. Scale bar = 20 μm. (C) Western blots of TFEB expression in cultured podocytes. After AGEs stimulation for 48 h, the nuclear TFEB and the cytosolic TFEB were respectively downregulated and upregulated, while the total TFEB was not changed significantly. Torin1 recovered nuclear TFEB expression, which was reduced in AGEs‐stimulated podocytes and did not affect total TFEB protein expression (mean ± SEM, n = 5). One‐way ANOVA, followed by post hoc Student–Newman–Keuls test, *P <0.05 versus control, #P <0.05 versus AGE.
**Figure S8. DNA electrophoretogram.** (A) Sonication effects were evaluated by agarose gel electrophoresis. The genomic DNA samples were ultrasonicated forming fragments with length of approximately 100–500 base pairs.
**Figure S9. Role of rapamycin on TFEB nuclear expression.** (A) Cultured podocytes were treated with 5 nM rapamycin or an equal volume of the dimethyl sulfoxide vehicle for 24 h. Rapamycin did not affect nuclear TFEB expression as measured by western blotting (means ± SEM, n = 3). Student's t‐test, *P <0.05 versus control.
**Figure S10. The proposed role of AGEs in suppressing podocyte autophagy.** (1) AGEs inhibit podocyte autophagy through mTOR activation. (2) AGEs suppress TFEB activity by inhibiting the nuclear translocation of TFEB and thus interrupting the translation of autophagy genes including Atg9b, LC3b, Lamp1 and Vps11. (3) AGEs‐induced mTOR activation decreases nuclear translocation of TFEB.

**Table S1.** Clinical characteristics in control subjects and patients with diabetic nephropathy

**Table S2.** SiRNA sequences

**Table S3.** Antibody information

**Table S4.** Primers used for ChIP assays

**Table S5.** Primers used for RT‐qPCR

## References

[1] Levin A , Tonelli M , Bonventre J , *et al* Global kidney health 2017 and beyond: a roadmap for closing gaps in care, research, and policy. Lancet 2017; 390: 1888–1917. 28434650 10.1016/S0140-6736(17)30788-2

[2] Rask‐Madsen C , King GL. Diabetes : podocytes lose their footing. Nature 2010; 468: 42–44. 21048754 10.1038/468042a

[3] Mizushima N , Komatsu M . Autophagy: renovation of cells and tissues. Cell 2011; 147: 728–741. 22078875 10.1016/j.cell.2011.10.026

[4] Mizushima N , Levine B , Cuervo AM , *et al* Autophagy fights disease through cellular self‐digestion. Nature 2008; 451: 1069–1075. 18305538 10.1038/nature06639 PMC2670399

[5] Zhou J , Tan SH , Codogno P , *et al* Dual suppressive effect of MTORC1 on autophagy: tame the dragon by shackling both the head and the tail. Autophagy 2013; 9: 803–805. 23439250 10.4161/auto.23965 PMC3669196

[6] Godel M , Hartleben B , Herbach N , *et al* Role of mTOR in podocyte function and diabetic nephropathy in humans and mice. J Clin Invest 2011; 121: 2197–2209. 21606591 10.1172/JCI44774 PMC3104746

[7] Inoki K , Mori H , Wang J , *et al* mTORC1 activation in podocytes is a critical step in the development of diabetic nephropathy in mice. J Clin Invest 2011; 121: 2181–2196. 21606597 10.1172/JCI44771 PMC3104745

[8] Yamagishi S , Inagaki Y , Okamoto T , *et al* Advanced glycation end product‐induced apoptosis and overexpression of vascular endothelial growth factor and monocyte chemoattractant protein‐1 in human‐cultured mesangial cells. J Biol Chem 2002; 277: 20309–20315. 11912219 10.1074/jbc.M202634200

[9] Feng Y , Yao Z , Klionsky DJ . How to control self‐digestion: transcriptional, post‐transcriptional, and post‐translational regulation of autophagy. Trends Cell Biol 2015; 25: 354–363. 25759175 10.1016/j.tcb.2015.02.002 PMC4441840

[10] Settembre C , Di Malta C , Polito VA , *et al* TFEB links autophagy to lysosomal biogenesis. Science 2011; 332: 1429–1433. 21617040 10.1126/science.1204592 PMC3638014

[11] Martina JA , Chen Y , Gucek M , *et al* MTORC1 functions as a transcriptional regulator of autophagy by preventing nuclear transport of TFEB. Autophagy 2012; 8: 903–914. 22576015 10.4161/auto.19653 PMC3427256

[12] Settembre C , Zoncu R , Medina DL , *et al* A lysosome‐to‐nucleus signalling mechanism senses and regulates the lysosome via mTOR and TFEB. EMBO J 2012; 31: 1095–1108. 22343943 10.1038/emboj.2012.32 PMC3298007

[13] Hartleben B , Godel M , Meyer‐Schwesinger C , *et al* Autophagy influences glomerular disease susceptibility and maintains podocyte homeostasis in aging mice. J Clin Invest 2010; 120: 1084–1096. 20200449 10.1172/JCI39492 PMC2846040

[14] Tagawa A , Yasuda M , Kume S , *et al* Impaired podocyte autophagy exacerbates proteinuria in diabetic nephropathy. Diabetes 2015; 65: 755–767. 26384385 10.2337/db15-0473

[15] Lenoir O , Jasiek M , Hénique C , *et al* Endothelial cell and podocyte autophagy synergistically protect from diabetes‐induced glomerulosclerosis. Autophagy 2015; 11: 1130–1145. 26039325 10.1080/15548627.2015.1049799 PMC4590611

[16] Wendt T , Tanji N , Guo J , *et al* Glucose, glycation, and RAGE: implications for amplification of cellular dysfunction in diabetic nephropathy. J Am Soc Nephrol 2003; 14: 1383–1395. 12707408 10.1097/01.asn.0000065100.17349.ca

[17] Tan X , Chen Y , Liang X , *et al* Lipopolysaccharide‐induced podocyte injury is mediated by suppression of autophagy. Mol Med Rep 2016; 14: 811–818. 27221629 10.3892/mmr.2016.5301

[18] Klionsky DJ , Abdelmohsen K , Abe A , *et al* Guidelines for the use and interpretation of assays for monitoring autophagy (3rd edition). Autophagy 2016; 12: 1‐222. 10.1080/15548627.2015.1100356 PMC4835977 26799652

[19] van den Berg JG , van den Bergh Weerman MA , Assmann KJ , *et al* Podocyte foot process effacement is not correlated with the level of proteinuria in human glomerulopathies. Kidney Int 2004; 66: 1901–1906. 15496161 10.1111/j.1523-1755.2004.00964.x

[20] Wei Q , Dong Z . HDAC4 blocks autophagy to trigger podocyte injury: non‐epigenetic action in diabetic nephropathy. Kidney Int 2014; 86: 666–668. 25265947 10.1038/ki.2014.142 PMC4181378

[21] Zeng C , Fan Y , Wu J , *et al* Podocyte autophagic activity plays a protective role in renal injury and delays the progression of podocytopathies. J Pathol 2014; 234: 203–213. 24870816 10.1002/path.4382

[22] Zheng F , Zeng YJ , Plati AR , *et al* Combined AGE inhibition and ACEi decreases the progression of established diabetic nephropathy in B6 db/db mice. Kidney Int 2006; 70: 507–514. 16775596 10.1038/sj.ki.5001578

[23] Obara I , Tochiki KK , Géranton SM , *et al* Systemic inhibition of the mammalian target of rapamycin (mTOR) pathway reduces neuropathic pain in mice. Pain 2011; 152: 2582–2595. 21917376 10.1016/j.pain.2011.07.025

[24] Tagawa A , Yasuda M , Kume S , *et al* Impaired podocyte autophagy exacerbates proteinuria in diabetic nephropathy. Diabetes 2016; 65: 755–767. 26384385 10.2337/db15-0473

[25] Wei C , Möller CC , Altintas MM , *et al* Modification of kidney barrier function by the urokinase receptor. Nat Med 2008; 14: 55–63. 18084301 10.1038/nm1696

[26] Hsu PP , Kang SA , Rameseder J , *et al* The mTOR‐regulated phosphoproteome reveals a mechanism of mTORC1‐mediated inhibition of growth factor signaling. Science 2011; 332: 1317–1322. 21659604 10.1126/science.1199498 PMC3177140

[27] Zhang C , Hou B , Yu S , *et al* HGF alleviates high glucose‐induced injury in podocytes by GSK3β inhibition and autophagy restoration. Biochim Biophys Acta 2016; 1863: 2690–2699. 27526674 10.1016/j.bbamcr.2016.08.004

[28] Sun J , Li ZP , Zhang RQ , *et al* Repression of miR‐217 protects against high glucose‐induced podocyte injury and insulin resistance by restoring PTEN‐mediated autophagy pathway. Biochem Biophys Res Commun 2017; 483: 318–324. 28017719 10.1016/j.bbrc.2016.12.145

[29] Li C , Siragy HM . (Pro)renin receptor regulates autophagy and apoptosis in podocytes exposed to high glucose. Am J Physiol Endocrinol Metab 2015; 309: E302–E310. 26081285 10.1152/ajpendo.00603.2014 PMC4525115

[30] Ma T , Zhu J , Chen X , *et al* High glucose induces autophagy in podocytes. Exp Cell Res 2013; 319: 779–789. 23384600 10.1016/j.yexcr.2013.01.018 PMC3628680

[31] Wei M , Li Z , Yang Z . Crosstalk between protective autophagy and NF‐κB signal in high glucose‐induced podocytes. Mol Cell Biochem 2014; 394: 261–273. 24957786 10.1007/s11010-014-2102-7

[32] Jin Y , Liu S , Ma Q , *et al* Berberine enhances the AMPK activation and autophagy and mitigates high glucose‐induced apoptosis of mouse podocytes. Eur J Pharmacol 2017; 794: 106–114. 27887947 10.1016/j.ejphar.2016.11.037

[33] Tamarat R , Silvestre JS , Huijberts M , *et al* Blockade of advanced glycation end‐product formation restores ischemia‐induced angiogenesis in diabetic mice. Proc Natl Acad Sci U S A 2003; 100: 8555–8560. 12805564 10.1073/pnas.1236929100 PMC166267

[34] Palmieri M , Impey S , Kang H , *et al* Characterization of the CLEAR network reveals an integrated control of cellular clearance pathways. Hum Mol Genet 2011; 20: 3852–3866. 21752829 10.1093/hmg/ddr306

[35] Medina DL , Fraldi A , Bouche V , *et al* Transcriptional activation of lysosomal exocytosis promotes cellular clearance. Dev Cell 2011; 21: 421–430. 21889421 10.1016/j.devcel.2011.07.016 PMC3173716

[36] Rega LR , Polishchuk E , Montefusco S , *et al* Activation of the transcription factor EB rescues lysosomal abnormalities in cystinotic kidney cells. Kidney Int 2016; 89: 862–873. 26994576 10.1016/j.kint.2015.12.045

[37] Peña‐Llopis S , Vega‐Rubin‐de‐Celis S , Schwartz JC , *et al* Regulation of TFEB and V‐ATPases by mTORC1. EMBO J 2011; 30: 3242–3258. 21804531 10.1038/emboj.2011.257 PMC3160667

[38] Marchand B , Arsenault D , Raymond‐Fleury A , *et al* Glycogen synthase kinase‐3 (GSK3) inhibition induces prosurvival autophagic signals in human pancreatic cancer cells. J Biol Chem 2015; 290: 5592–5605. 25561726 10.1074/jbc.M114.616714 PMC4342473

[39] Palmieri M , Pal R , Nelvagal HR , *et al* mTORC1‐independent TFEB activation via Akt inhibition promotes cellular clearance in neurodegenerative storage diseases. Nat Commun 2017; 8: 14338. 28165011 10.1038/ncomms14338 PMC5303831

[40] Mundel P , Reiser J , Zúñiga MBA , *et al* Rearrangements of the cytoskeleton and cell contacts induce process formation during differentiation of conditionally immortalized mouse podocyte cell lines. Exp Cell Res 1997; 236: 248–258. 9344605 10.1006/excr.1997.3739

[41] Li R , Zhang L , Shi W , *et al* NFAT2 mediates high glucose‐induced glomerular podocyte apoptosis through increased Bax expression. Exp Cell Res 2013; 319: 992–1000. 23340267 10.1016/j.yexcr.2013.01.007

[42] Livak KJ , Schmittgen TD . Analysis of relative gene expression data using real‐time quantitative PCR and the 2(−Delta Delta C(T)) method. Methods 2001; 25: 402–408. 11846609 10.1006/meth.2001.1262

[43] Yozai K , Shikata K , Sasaki M , *et al* Methotrexate prevents renal injury in experimental diabetic rats via anti‐inflammatory actions. J Am Soc Nephrol 2005; 16: 3326–3338. 16177002 10.1681/ASN.2004111011

[44] Lin JS , Shi Y , Peng H , *et al* Loss of PTEN promotes podocyte cytoskeletal rearrangement, aggravating diabetic nephropathy. J Pathol 2015; 236: 30–40. 25641678 10.1002/path.4508 PMC4398628

