# Supplementary material for: Advanced glycation end‐products suppress autophagic flux in podocytes by activating mammalian target of rapamycin and inhibiting nuclear translocation of transcription factor EB
Source: J Pathol. 2018 Apr 30;245(2):235–48. doi: 10.1002/path.5077 (PMC5969319; doi:10.1002/path.5077)
Supplement: Supplementary file 1 — Appendix S1. Supplementary materials and methods [file PATH-245-235-s001.docx]

**Supplementary materials and methods**

Reference numbers refer to the main text reference list

**Cell culture**

The conditionally immortalized mouse podocyte cell line was a kind gift from Dr Jochen Reiser and cultured as described previously [40]. Cells were grown at 33°C in RPMI-1640 medium (Gibco BRL, Gaithersburg, MD, USA) with 10% FBS (Gibco BRL) and recombinant IFN-γ (ProSpec, Tany Technogene, Ness Ziona, Israel). To induce differentiation, podocytes were reseeded and cultured at 37°C in 100 cm^2^ culture dishes coated with collagen type-I (BD Bioscience, Bedford, MA, USA) and in RPMI-1640 medium supplemented with 5% FBS but without IFN-γ (growth restrictive conditions) for 10–13 days. Differentiated podocytes were confirmed by expression of synaptopodin (a podocyte differentiation marker).

**Western blotting**

Whole-cell and nuclear proteins were prepared as described previously [41]. Proteins (10–30 µg) were loaded onto 7.5–10% sodium dodecyl sulfate-polyacrylamide gels and then transferred to PVDF membranes (Millipore, Billerica, MA, USA). After blocking non-specific binding with 5% non-fat dry milk for 1 h at room temperature, PVDF membranes were incubated overnight at 4°C with primary antibodies (supplementary material, Table S3). Then PVDF membranes were incubated with secondary antibodies (supplementary material, Table S3) at room temperature for 1 h. Finally, PVDF membranes were treated with ECL reagents (Advansta, Menlo Park, CA, USA). Protein bands were visualized using an automatic imager (General Electric, Fairfield, CT, USA).

**Co-immunoprecipitation (Co-IP) assays**

Co-IP experiments were undertaken using a Dynabeads Protein G Immunoprecipitation kit (Invitrogen, Carlsbad, CA, USA) according to the manufacturer’s instructions. In brief, 10 μg of anti-TFEB antibody (Ab) (supplementary material, Table S3) or control IgG was incubated with 50 μl of Dynabeads protein G for 2 h. The tube was placed on the magnet and the supernatant removed. Then 400 μg of cell lysate was used as input material and incubated with the Dynabeads–Ab complex overnight at 4°C with rotation. The Dynabeads–Ab–antigen complex was washed with 100 μl of washing buffer and eluted with 2× standard Laemmli buffer for western blotting.

**Reverse transcription–quantitative PCR (RT-qPCR)**

Total RNA was extracted using a TRIzol RNA isolation system (Invitrogen, Carlsbad, CA, USA) and reverse-transcribed into cDNAs using a PrimeScript™ RT Reagent kit according to the manufacturer’s instructions (TaKaRa Biotechnology, Shiga, Japan). Then cDNAs were subjected to quantitative PCR using a Power SYBR Green PCR Master Mix (TaKaRa Biotechnology). Data were calculated using the 2^−ΔΔCq^ method with GAPDH as the reference gene as previously described [42]. Primer sequences are listed in the supplementary material, Table S5.

**Immunofluorescence staining**

Cultured podocytes seeded on coverslips in six-well plates or frozen cryostat sections were fixed with 4% paraformaldehyde at room temperature for 15 min and then permeabilized with 0.1% Triton X-100 for 10 min. After blocking non-specific binding with 5% bovine serum albumin for 30 min at room temperature, cells or sections were incubated overnight at 4°C with primary antibodies (supplementary material, Table S3). After washing three times with phosphate-buffered saline for 5 min, cells or sections were incubated in the dark with secondary antibodies (supplementary material, Table S3) for 1 h at room temperature. Cells or sections were then stained with DAPI (Sigma-Aldrich) for 10 min at room temperature. Images were prepared using confocal laser scanning microscopy (KS 400; Zeiss, Postfach, Germany) and analyzed using Image-Pro Plus v6.0 (Media Cybernetics, Rockville, MD, USA). All images were analyzed by two investigators blinded to the identity of the samples.

**Renal histology**

Paraffin-embedded tissues were sectioned at 4 μm and stained with hematoxylin and eosin (H&E) or periodic acid–Schiff (PAS) stain for light microscopy. Mesangial expansion was evaluated by the mesangial matrix index, which was calculated as the ratio of the PAS-positive and nucleus-free mesangial area to the glomerular tuft area and reported as a percentage as previously described [43,44]. The mesangial and glomerular tuft areas were measured using Image-Pro Plus v6.0 (Media Cybernetics). Thirty glomeruli were selected randomly from each experimental group for the measurement of mesangial matrix area.
