# Supplementary material for: Advanced glycation end‐products suppress autophagic flux in podocytes by activating mammalian target of rapamycin and inhibiting nuclear translocation of transcription factor EB
Source: J Pathol. 2018 Apr 30;245(2):235–48. doi: 10.1002/path.5077 (PMC5969319; doi:10.1002/path.5077)
Supplement: Supplementary file 2 — Figure S1. Role of AGEs on LC3II expression. (A) Cultured podocytes were treated with BSA or AGE‐BSA (AGE) for the indicated time. Western blotting indicated that AGE inhibited LC3II expression at 72 h (mean ± SEM, n = 3) One way ANOVA, followed by post hoc Student–Newman–Keuls test. *P < 0.05 versus control. Figure S2. AGEs block autophagosome formation. AGEs decreased chloroquine (CQ)‐induced LC3II accumulation as shown by western blotting (A) and reduced CQ‐induced autophagosome accumulation on TEM (B) (means ± SEM, n = 3, and 27–30 images from each group). One‐way ANOVA, followed by post hoc Student–Newman–Keuls test. *P <0.05 versus control, #P <0.05 versus CQ. Figure S3. Torin1 reduces mTOR activity. (A) Western blot assays show reduction of the p‐p70s6k to p70s6k ratio at 3 h in cultured podocytes by various Torin1 (TR) concentrations (mean ± SEM, n = 3). One‐way ANOVA, followed by post hoc Student–Newman–Keuls test. *P <0.05 versus control. (B) Western blot assays of p‐p70s6k and p70s6k expression from the renal cortex showed that the p‐p70s6k to p70s6k ratio was increased in db/db mice (mean ± SEM, n = 4). Two‐way ANOVA, followed by post hoc Student–Newman–Keuls test. *P <0.05 versus control, #P <0.05 versus db/db mice. Figure S4. Efficiency of TFEB interference and podocyte injury by TFEB knockdown. (A) Cultured podocytes were transfected with three siRNAs designed to target TFEB or scrambled siRNA. RT‐qPCR showed that TFEB mRNA expression was significantly downregulated by TFEB siRNA but not TFEB siRNA‐2 or TFEB siRNA‐3 (means ± SEM, n = 3). One‐way ANOVA, followed by post hoc Student–Newman–Keuls test, *P <0.05 versus control. (B) Western blot assay results showed that TFEB siRNA significantly reduced TFEB protein expression (mean ± SEM, n = 3). One‐way ANOVA, followed by post hoc Student–Newman–Keuls test. *P <0.05 versus control. (C) Western blot assays showed that TFEB siRNA significantly reduced nuclear TFEB expression (mean ± SEM, n = 3). One‐way AN [file PATH-245-235-s002.pdf]

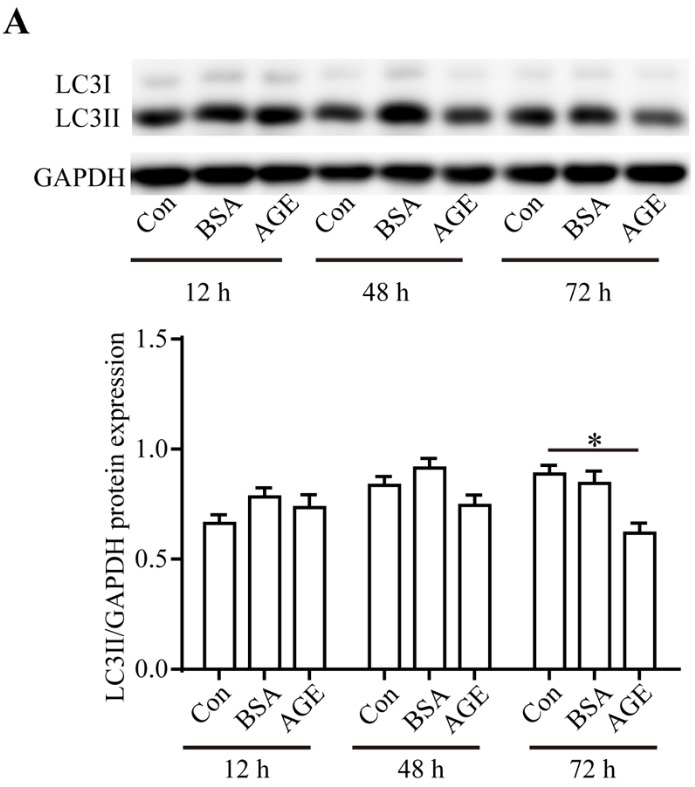

Figure S1. Role of AGEs in LC3II expression. (A) Cultured podocytes were treated with BSA or AGE-BSA (AGE) for the indicated time. Western blotting indicated that AGE inhibited LC3II expression at 72 h (mean ± SEM, n = 3) One way ANOVA, followed by *post hoc* Student–Newman–Keuls test. $^{*}P < 0.05$ versus control.

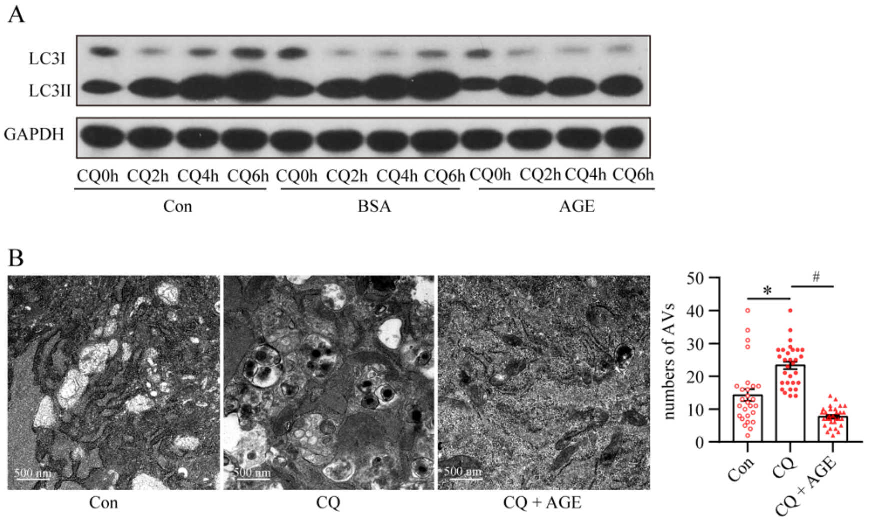

Figure S2. AGEs block autophagosome formation. AGEs decreased chloroquine (CQ)-induced LC3II accumulation as shown by western blotting (A) and reduced CQ-induced autophagosome accumulation on TEM (B) (means ± SEM, n = 3, and 27–30 images from each group). One-way ANOVA, followed by *post hoc* Student–Newman–Keuls test. $^{*}P$ <0.05 versus control, $^{\#}P$ <0.05 versus CQ.

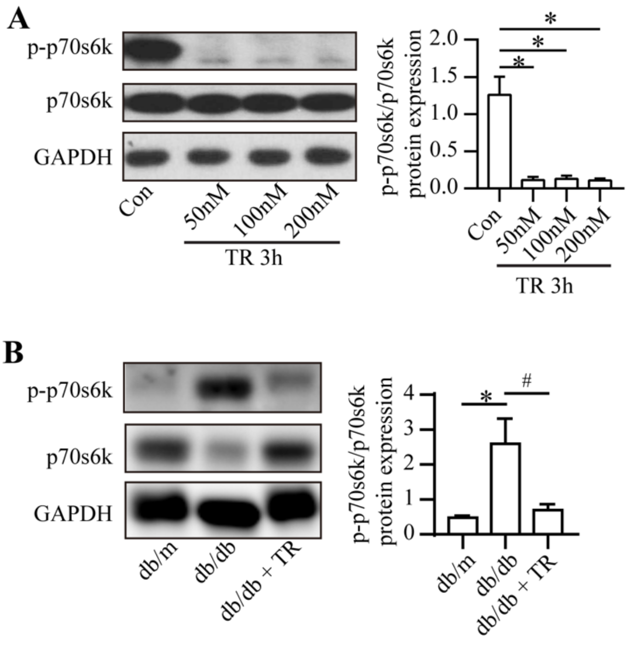

Figure S3 Torin1 reduces mTOR activity. (A) Western blot assays show reduction of the p-p70s6k to p70s6k ratio at 3 h in cultured podocytes by various Torin1 (TR) concentrations (mean ± SEM, n = 3). One-way ANOVA, followed by *post hoc* Student–Newman–Keuls test. $^{*}P$ <0.05 versus control. (B) Western blot assays of p-p70s6k and p70s6k expression from the renal cortex showed that the p-p70s6k to p70s6k ratio was increased in db/db mice (mean ± SEM, n = 4). Two-way ANOVA, followed by *post hoc* Student–Newman–Keuls test. $^{*}P$ <0.05 versus control, $^{\#}P$ <0.05 versus db/db mice.

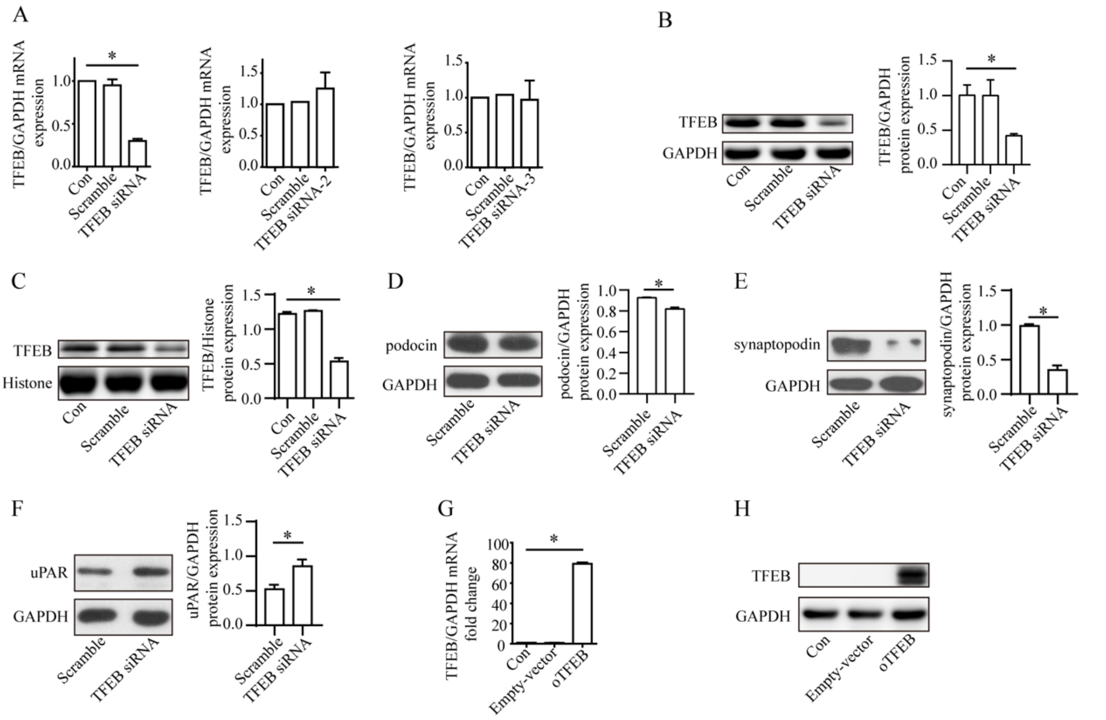

Figure S4. Efficiency of TFEB interference and podocyte injury by TFEB knockdown. (A) Cultured podocytes were transfected with three siRNAs designed to target TFEB or scrambled siRNA. RT-qPCR showed that TFEB mRNA expression was significantly downregulated by TFEB siRNA but not TFEB siRNA-2 or TFEB siRNA-3 (means ± SEM, n = 3). One-way ANOVA, followed by *post hoc* Student–Newman–Keuls test, $^{*}P$ <0.05 versus control. (B) Western blot assay results showed that TFEB siRNA significantly reduced TFEB protein expression (mean ± SEM, n = 3). One-way ANOVA, followed by *post hoc* Student–Newman–Keuls test. $^{*}P$ <0.05 versus control. (C) Western blot assays showed that TFEB siRNA significantly reduced nuclear TFEB expression (mean ± SEM, n = 3). One-way ANOVA, followed by *post hoc* Student–Newman–Keuls test, $^{*}P$ <0.05 versus control. (D–F) TFEB siRNA significantly downregulated the expression of podocin (D) and synaptopodin (E) proteins, which are markers of intact podocytes, and (F) upregulated the expression of urokinase-type plasminogen activator receptor (uPAR) a marker of podocyte injury. (mean ± SEM, n = 3) Student's *t*-test, $^{*}P$ <0.05 versus scrambled siRNA. (G) Cultured podocytes were transfected with and empty vector control or flag-TFEB adenovirus (oTFEB). Expression of TFEB mRNA was significantly increased by oTFEB (mean ± SEM, n = 3). One-way ANOVA, followed by *post hoc* Student–Newman–Keuls test, $^{*}P$ <0.05 versus control. (H) Western blot assays show that TFEB protein expression was upregulated by oTFEB.

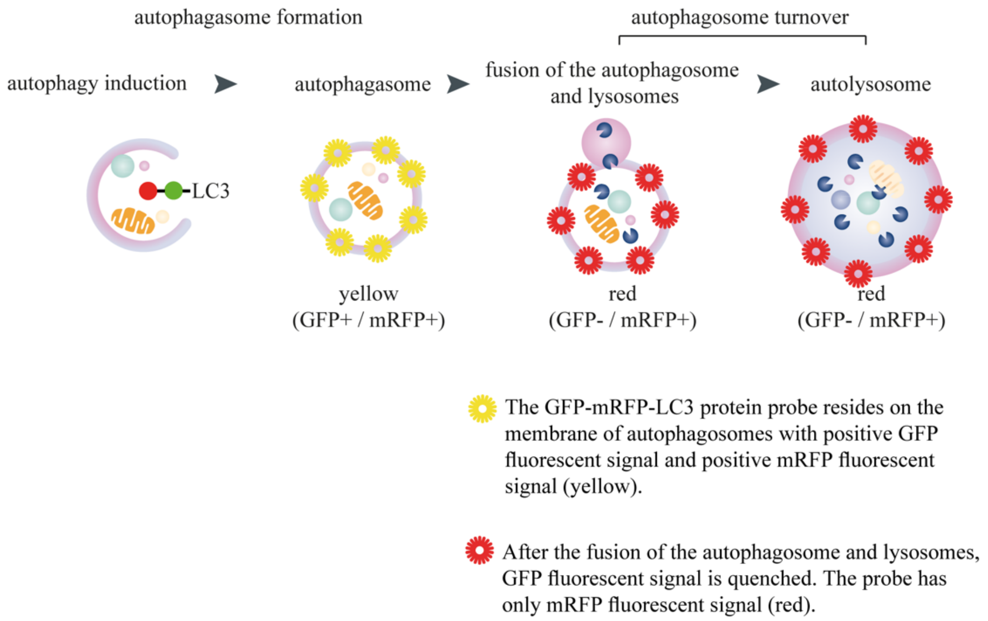

Figure S5. The principle of the GFP-mRFP-LC3 fluorescent probe.

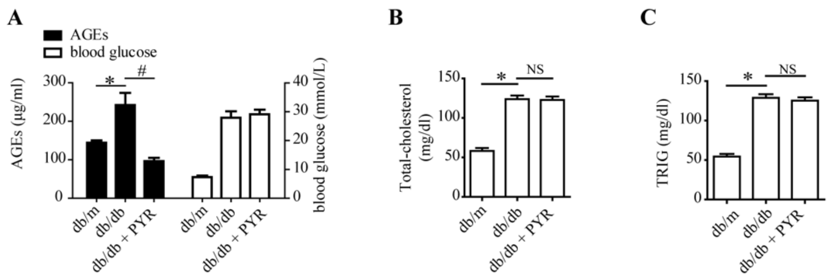

Figure S6. Effect of pyridoxamine (PYR) on blood levels of glucose, AGEs, and lipid parameters in experimental mice. Serum AGEs (A), blood glucose (A), total cholesterol (B) and triglycerides (TRIGs) (C) were increased in db/db mice compared with db/m control mice. PYR reduced AGEs without affecting blood glucose, total cholesterol and TRIGs (mean ± SEM, n = 3). Two-way ANOVA, followed by *post hoc* Student–Newman–Keuls test, $^{*}P$ <0.05 versus db/m mice, $^{\#}P$ <0.05 versus db/db mice. NS: no significance.

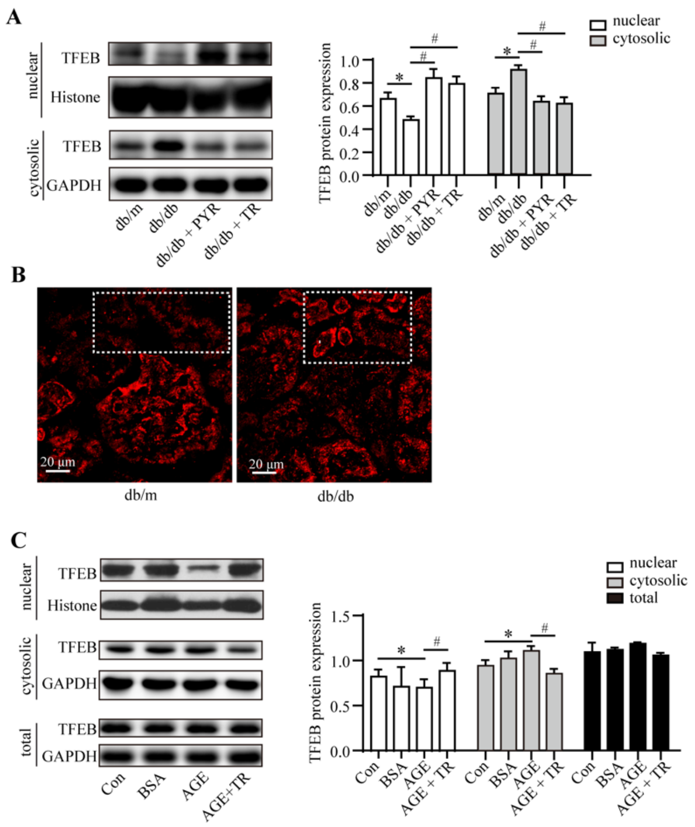

Figure S7. Change in TFEB expression in db/db mice and AGEs-induced podocytes. (A) TFEB expression in the nuclear and cytosolic fractions of mouse renal cortex was assayed by western blotting. Both PYR or torin1 reduced the expression of nuclear TFEB in db/db mice and increased cytosolic expression (mean ± SEM, n = 4). One-way ANOVA, followed by *post hoc* Student–Newman–Keuls test, $^{*}P < 0.05$ versus db/m mice, $^{\#}P < 0.05$ versus db/db mice. (B) Immunofluorescence staining of TFEB (red) in the renal cortex of db/db mice and db/m mice showed that TFEB was expressed not only in podocytes but also in other renal resident cells (dashed frame). TFEB expression in the renal tubules tended to increase under diabetic conditions. Scale bar = 20 μm. (C) Western blots of TFEB expression in cultured podocytes. After AGEs stimulation for 48 h, the nuclear TFEB and the cytosolic TFEB were respectively downregulated and upregulated, while the total TFEB was not changed significantly. Torin1 recovered nuclear TFEB expression, which was reduced in AGEs-stimulated podocytes and did not affect total TFEB protein expression (mean ± SEM, n = 5). One-way ANOVA, followed by *post hoc* Student–Newman–Keuls test, $^{*}P < 0.05$ versus control, $^{\#}P < 0.05$ versus AGE.

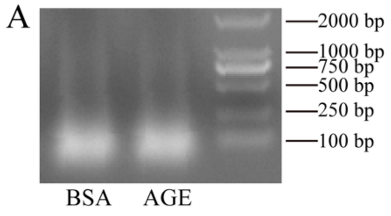

Figure S8. DNA electrophoretogram. (A) Sonication effects were evaluated by agarose gel electrophoresis. The genomic DNA samples were ultrasonicated forming fragments with length of approximately 100–500 base pairs.

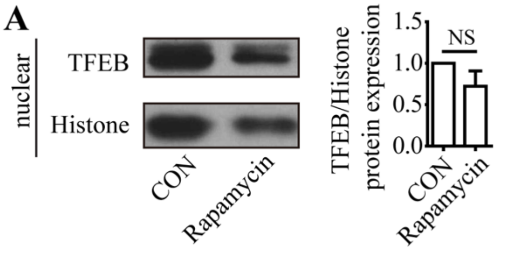

Figure S9. Role of rapamycin in TFEB nuclear expression. (A) Cultured podocytes were treated with 5 nM rapamycin or an equal volume of the dimethyl sulfoxide vehicle for 24 h. Rapamycin did not affect nuclear TFEB expression as measured by western blotting (means ± SEM, n = 3). Student's *t*-test, $^{*}P < 0.05$ versus control.

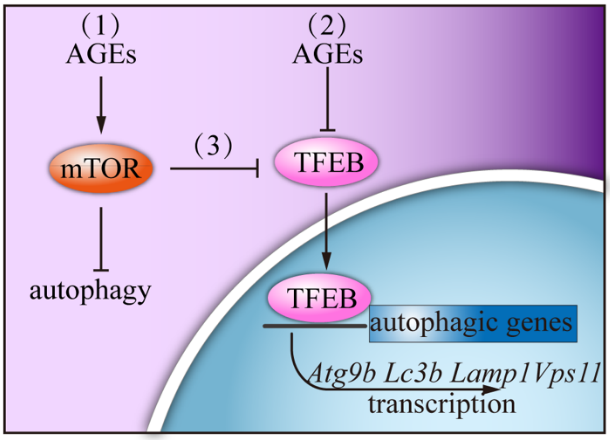

Figure S10. The proposed role of AGEs in suppressing podocyte autophagy. (1) AGEs inhibit podocyte autophagy through mTOR activation. (2) AGEs suppress TFEB activity by inhibiting the nuclear translocation of TFEB and thus interrupting the translation of autophagy genes including *Atg9b*, *LC3b*, *Lamp1* and *Vps11*. (3) AGEs-induced mTOR activation decreases nuclear translocation of TFEB.
