# Supplementary material for: Advanced glycation end‐products suppress autophagic flux in podocytes by activating mammalian target of rapamycin and inhibiting nuclear translocation of transcription factor EB
Source: J Pathol. 2018 Apr 30;245(2):235–48. doi: 10.1002/path.5077 (PMC5969319; doi:10.1002/path.5077)
Supplement: Supplementary file 3 — Table S1. Clinical characteristics in control subjects and patients with diabetic nephropathy [file PATH-245-235-s004.doc]

**Table S1.** Clinical characteristics in control subjects and patients with diabetic nephropathy

|  | **Age (years)** | **Weight(kg)** | **Height (cm)** | **BMI** | **BP**  **(mmHg)** | **FG**  **(mmol/l)** | **Crea**  **(μmol/l)** | **BUN (mmol/l)** | **UACR (mg/g)** | **UPCR (mg/g)** | **Maximum of** **HbA1c (%)** | **Mean of HbA1c (%)** | **ALB**  **(g/l)** | **TRIG (mmol/l)** | **CHOL**  **(mmol/l)** |
| --- | --- | --- | --- | --- | --- | --- | --- | --- | --- | --- | --- | --- | --- | --- | --- |
| DN1 | 43 | 64.5 | 164 | 24 | 152/64 | 4.8 | 77.8 | 6.6 | 2454.4 | 6176.0 | 7.3 | 7.2 | 26.1 | 1.7 | 6.5 |
| DN2 | 48 | 63.2 | 159 | 25 | 146/96 | 8.4 | 332.7 | 16.1 | 2369.4 | 5193.3 | 6.5 | 5.9 | 40.1 | 3.9 | 4.5 |
| DN3 | 54 | 82.0 | 169 | 29 | 169/106 | 6.8 | 72.9 | 4.8 | 141.6 | 345.9 | 10.4 | 6.1 | 42.5 | 1.9 | 3.3 |
| DN4 | 43 | 59.4 | 165 | 22 | 145/77 | 9.4 | 121.1 | 9.0 | 5077.3 | 8862.3 | 9.3 | 5.7 | 35.4 | 1.6 | 3.0 |
| DN5 | 52 | 75.0 | 177 | 24 | 152/91 | 14.8 | 329.7 | 10.8 | 5753.4 | 11187.1 | 8.7 | 7.8 | 27.9 | 7.9 | 10.0 |
| CON1 | 60 | 63.4 | 172 | 21 | 149/93 | 4.5 | 61.4 | 4.2 | 5.6 | 98.7 | 4.4 | - | 47.0 | 1.8 | 3.5 |
| CON2 | 34 | 55.1 | 165 | 20 | 153/89 | 3.9 | 52.0 | 5.3 | 10.1 | 53.3 | 5.2 | - | 39.0 | 0.9 | 3.2 |
| CON3 | 45 | 66.9 | 169 | 23 | 143/80 | 4.8 | 42.0 | 6.3 | 9.7 | 87.6 | 4.7 | - | 44.2 | 0.7 | 3.2 |
| CON4 | 57 | 72.3 | 179 | 23 | 138/65 | 5.1 | 83.0 | 7.6 | 16.8 | 96.3 | 5.5 | - | 47.0 | 1.7 | 5.3 |

DN: diabetic nephropathy; BMI: body mass index; BP: blood pressure; FG: fasting glucose; Crea: serum creatinine; BUN: blood urea nitrogen; UACR: urine albumin to creatinine ratio; UPCR: urine protein to creatinine ratio; HbA1c: glycated hemoglobin; maximum of HbA1c: the maximal value detected from 3 months before renal biopsy to the date of renal biopsy; mean of HbA1c: the mean of HbA1c from 3 months before renal biopsy to the date of renal biopsy; ALB: serum albumin; TRIG: triacylglycerol; CHOL: total cholesterol.
