# Supplementary material for: Advanced glycation end‐products suppress autophagic flux in podocytes by activating mammalian target of rapamycin and inhibiting nuclear translocation of transcription factor EB
Source: J Pathol. 2018 Apr 30;245(2):235–48. doi: 10.1002/path.5077 (PMC5969319; doi:10.1002/path.5077)
Supplement: Supplementary file 4 — Table S2. SiRNA sequences [file PATH-245-235-s007.docx]

**Table S2.** SiRNA sequences

| Name | Sequence |
| --- | --- |
| TFEB siRNA | CCATGGCCATGCTACATAT |
| TFEB siRNA-2 | GCAGGCTGTCATGCATTAT |
| TFEB siRNA-3 | CCAAGAAGGATCTGGACTT |
