# Supplementary material for: Advanced glycation end‐products suppress autophagic flux in podocytes by activating mammalian target of rapamycin and inhibiting nuclear translocation of transcription factor EB
Source: J Pathol. 2018 Apr 30;245(2):235–48. doi: 10.1002/path.5077 (PMC5969319; doi:10.1002/path.5077)
Supplement: Supplementary file 5 — Table S3. Antibody information [file PATH-245-235-s003.docx]

**Table S3.** Antibody information

**Primary antibodies:**

| **Name** | **Species** | **Supplier** | **Dilution** | **Application** |
| --- | --- | --- | --- | --- |
| Anti-LC3A/B | R | Cell Signaling Technology (CST), Danvers, MA, USA | 1:1000 | WB |
| Anti-p62 | R | CST, Danvers, MA, USA | 1:1000 | WB |
| Anti-beclin1 | R | CST, Danvers, MA, USA | 1:1000 | WB |
| Anti-phospho-p70s6k | R | CST, Danvers, MA, USA | 1:1000 | WB |
| Anti-Histone H3 | R | CST, Danvers, MA, USA | 1:3000 | WB |
| Anti-TFEB | R | Bethyl, Montgomery, TX, USA | 1:1000 | WB |
| Anti-GAPDH | R | Bioworld Technology, Nanjing, China | 1:3000 | WB |
| Anti-mTOR | M | Abcam, Cambridge, MA, USA | 1:500 | WB |
| Anti-p70s6k | R | Abcam, Cambridge, MA, USA | 1:1000 | WB |
| Anti-synaptopodin | G | Santa Cruz, CA, USA | 1:1000 | WB |
| Anti-podocin | R | Sigma-Aldrich, St Louis, MO, USA | 1:2000 | WB |
| Anti-mTOR | M | Abcam, Cambridge, MA, USA | 1:1000 | WB |
| Anti-TFEB | G | Abcam, Cambridge, MA, USA | 1:100 | ChIP |
| Anti-synaptopodin | G | Santa Cruz, CA, USA | 1:100 | IF |
| Anti-WT-1 | G | Santa Cruz, CA, USA | 1:100 | IF |
| Anti-TFEB | R | Bethyl, Montgomery, TX, USA | 1:200 | IF |
| Anti- phospho-p70s6k | R | CST, Danvers, MA, USA, | 1:100 | IF |
| Anti-LC3A/B | R | CST, Danvers, MA, USA, | 1:250 | IF |
| Anti-TFEB | G | Abcam, Cambridge, MA, USA | 1:200 | IF |
| Anti-TFEB | R | Bethyl, Montgomery, TX, USA | 10 g/mg lysate | Co-IP |

WB: western blot; ChIP: chromatin immunoprecipitation; IF: immunofluorescent staining;
Co-IP: co-immunoprecipitation; R: rabbit; G: goat; M: mouse.

**Secondary antibodies:**

| **Name** | **Supplier** | **Dilution** | **Application** |
| --- | --- | --- | --- |
| Anti-rabbit IgG | Thermo Fisher Scientific, Rockford, IL, USA | 1: 3000 | WB |
| Anti-mouse IgG | CST, Danvers, MA, USA | 1: 3000 | WB |
| Anti-goat IgG | CST, Danvers, MA, USA | 1: 3000 | WB |
| FITC-donkey anti-goat IgG 488 | Invitrogen by Thermo Fisher | 1:200 | IF |
| Goat anti-rabbit Alexa Fluor 555 | CST, Danvers, MA, USA | 1:250 | IF |
| Goat anti-rabbit Alexa Fluor 488 | Invitrogen by Thermo Fisher | 1:250 | IF |
| Goat anti-mouse Alexa Fluor 555 | CST, Danvers, MA, USA | 1:250 | IF |

WB: western blot; IF: immunofluorescent staining; R: rabbit; G: goat; M: mouse.
