# Supplementary material for: Advanced glycation end‐products suppress autophagic flux in podocytes by activating mammalian target of rapamycin and inhibiting nuclear translocation of transcription factor EB
Source: J Pathol. 2018 Apr 30;245(2):235–48. doi: 10.1002/path.5077 (PMC5969319; doi:10.1002/path.5077)
Supplement: Supplementary file 6 — Table S4. Primers used for ChIP assays [file PATH-245-235-s005.docx]

**Table S4.** Primers used for ChIP assays

| **Target** | **Forward primer** | **Reverse primer** |
| --- | --- | --- |
| *Atg9b* | TCCCGCTACTCTGTCTCCTC | TTCCTACAATTGCACTCCCA |
| *Map1lc3b*  (*Lc3b*) | GCTTCCGCCACGCCTGTCAT | GTCTCAGTCCGCAGCCGAGT |
| *Lamp1* | GGGGTGGGGAGAGGGCAAGA | CCCGTGGACCGCCAGCTTAC |
| *Vps11* | AAAGAGCATTAAGGAGCTACGG | GAAAGGCGAGTCATGTGACAGT |
