# Supplementary material for: Advanced glycation end‐products suppress autophagic flux in podocytes by activating mammalian target of rapamycin and inhibiting nuclear translocation of transcription factor EB
Source: J Pathol. 2018 Apr 30;245(2):235–48. doi: 10.1002/path.5077 (PMC5969319; doi:10.1002/path.5077)
Supplement: Supplementary file 7 — Table S5. Primers used for RT‐qPCR [file PATH-245-235-s006.docx]

**Table S5.** Primers used for RT-qPCR

| **Target** | **Forward primer** | **Reverse primer** |
| --- | --- | --- |
| *Map1lc3a* (*Lc3a)* | GACCGCTGTAAGGAGGTGC | CTTGACCAACTCGCTCATGTTA |
| *Map1lc3b*  (*Lc3b*) | TTATAGAGCGATACAAGGGGGAG | CGCCGTCTGATTATCTTGATGAG |
| *Atg9a* | CAGTTTGACACTGAATACCAGCG | AATGTGGTGCCAAGGTGATTT |
| *Atg9b* | ATCAGCGGAATGGCTTTGC | TGGTTGGTTGTTGAAGAGAACAT |
| *Beclin1* | ATGGAGGGGTCTAAGGCGTC | TCCTCTCCTGAGTTAGCCTCT |
| *Uvrag* | ACATCGCTGCTCGGAACATT | CTCCACGTCGGATTCAAGGAA |
| *Lamp1* | CAGCACTCTTTGAGGTGAAAAAC | ACGATCTGAGAACCATTCGCA |
| *Vps11* | AAAAGAGAGACGGTGGCAATC | AGCCCAGTAACGGGATAGTTG |
| *TFEB* | CCACCCCAGCCATCAACAC | CAGACAGATACTCCCGAACCTT |
| *GAPDH* | AGGTCGGTGTGAACGGATTTG | TGTAGACCATGTAGTTGAGGTCA |
